# Surprising Radiolytic Stability of 8-Thiomethyladenine in an Aqueous Solution

**DOI:** 10.1021/acs.jpcb.4c01033

**Published:** 2024-04-05

**Authors:** Magdalena Datta, Adrian Szczyrba, Magdalena Zdrowowicz, Dariusz Wyrzykowski, Olga Ciupak, Sebastian Demkowicz, Farhad Izadi, Stephan Denifl, Janusz Rak

**Affiliations:** † Faculty of Chemistry, University of Gdańsk, Wita Stwosza 63, Gdańsk 80-308, Poland; ‡ Department of Organic Chemistry, Faculty of Chemistry, Gdańsk University of Technology, Narutowicza 11/12, 80-233 Gdańsk, Poland; § Institut für Ionenphysik und Angewandte Physik and Center for Biomolecular Sciences Innsbruck, Universität Innsbruck, Technikerstrasse 25, A-6020 Innsbruck, Austria

## Abstract

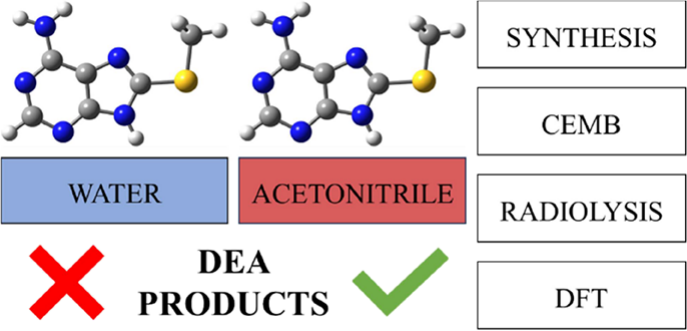

8-Thiomethyladenine (ASCH_3_), a potentially radiosensitizing modified nucleobase, has been synthesized in a reaction between 8-thioadenine and methyl iodide. Despite favorable dissociative electron attachment (DEA) characteristics, the radiolysis of an aqueous solution of ASCH_3_ with a dose of X-ray amounting to as much as 300 Gy leads to no effects. Nevertheless, crossed electron-molecule beam experiments in the gas phase on ASCH_3_ confirm the theoretical findings regarding the stability of its radical anion, namely, the most abundant reaction channel is related to the dissociation of the S-CH_3_ bond in the respective anion. Furthermore, electron-induced degradation of ASCH_3_ has been observed in aprotic acetonitrile, which is strong evidence for the involvement of proton transfer (PT) in stabilizing the radical anion in an aqueous solution. These findings demonstrate that PT in water can be the main player in deciding the radiosensitizing properties of modified nucleobases/nucleosides.

## 1 Introduction

Employing ionizing radiation (IR) radiotherapy is one of the most suitable modalities used against cancer.^1^ Its effectiveness is based on the interaction between cellular DNA and products of water radiolysis, mainly hydroxyl radicals. However, in solid tumors (which constitute ca. 80% of all tumor cases) the level of oxygen is significantly lower (hypoxia) than that in normoxic cells, which diminishes the effectiveness of IR due to the so-called oxygen effect, i.e. hypoxia makes the cells threefolds less sensitive to IR compared to those with physiological level of oxygen.^2^ One should remember that IR itself may introduce mutations to the genomic DNA that in the worst case lead to secondary cancer. Taking into account the fact that cells in surrounding tissues are usually at physoxia, the risk of their damage seems to be higher than that for tumor cells. To overcome this problem, one can introduce specific compounds to radiotherapy that sensitize cancer cells to IR despite their lower level of dissolved oxygen. Modified nucleosides (NBs) belong to hypoxic cell radiosensitizers that utilize the second most abundant product of water radiolysis, i.e. hydrated electrons.^3^ It is worth noticing that after incorporation into DNA and attachment of electrons, NBs form radical anions that are kinetically and thermodynamically unstable.^4^ Indeed, a well-known 5-bromo-2′-deoxyuridine (BrdU), a representative of hypoxic cell radiosensitizers, dissociates after electron attachment, forming the bromide anion and a reactive uracil-5-yl radical.^5^ The latter stabilizes in a series of secondary reactions, ultimately leading to a DNA strand break. Hence, the transformation of a native nucleoside in a derivative with high electron affinity and low stability in the radical anion form is the main idea behind radiosensitizing NBs.^6^

Radiosensitizing NBs^7^ proposed so far are mainly derivatives of uridine. Analogs of purine nucleosides are quite scarce. To this end, one can enumerate mainly bromoderivatives of adenosine and guanosine.^8^ Low-energy electron (LEE) attachment studies were carried out with 8-bromoadenine^9−12^ and 2-fluoroadenine.^13^ Similarly, LEE-induced damage to short single-stranded oligonucleotides labeled with 8-bromoadenine or 8-bromoguanine was studied under ultrahigh vacuum^14,15^ while the impact of solvated electrons in an aqueous solution on those sequences was elucidated in radiolytic investigations.^16^ Since there are no fundamental reasons against radiosensitizing purine nucleosides with other modifications than halogens and working out such systems would extend quite a limited radiosensitizer pool, we decided to synthesize potentially radiosensitizing derivatives of adenosine, 8-thiomethyladenine (ASCH_3_), and characterize its properties in the gas phase and aqueous solution. Indeed, the gas phase experiments (crossed electron-molecule beam (CEMB) measurements) coupled to quantum-chemical (QM) calculations confirmed the propensity to dissociative electron attachment (DEA) of the above-mentioned derivative. However, the aqueous phase studies did not demonstrate ASCH_3_ susceptibility to damage by electron attachment. In the discussion below, we suggest that this unexpected behavior is probably caused by the protonation of the ASCH_3_ nucleobase in an aqueous solution. This hypothesis was confirmed by radiolysis in aprotic acetonitrile. The experimental results followed by QM calculations at the density functional theory level seem to confirm our suppositions.

Adenine, a purine nucleobase, does not undergo chemical modifications as easily as uracil. On the other hand, it was demonstrated that 8-brominated guanosine is prone to the DEA process.^17^ Analysis of chemical modification possibilities suggests adenine substitution at the C8 position is the most facile. Therefore, we first obtained 8-bromoadenine (8BrA) from adenine, then transformed 8BrA into 6-amino-7(*H*)-purine-8(9*H*)-thione (8SA), and finally converted 8SA to 8-methylthioadenine.^18^ Designing this potentially radiosensitive derivative, i.e., a system of high susceptibility to DEA, we considered the Hammett inductive constant of a substituent (for the −SCH_3_ group it amounts to 0.25,^19^ which implies that such a modification of adenine should lead to thermodynamically stable radical anion^20^). Additionally, the S–C bond energy is similar to that of the C–Br bond, suggesting, thus, a low-barrier dissociation,^21^ a second requirement for efficient DEA. Taking into account the above reasoning, it appears that ASCH_3_ should possess significant radiosensitizing features.

## 2 Methods

### 2.1 Synthesis

#### 2.1.1 Synthesis of 8-Bromoadenine **1**

**Figure 1:**
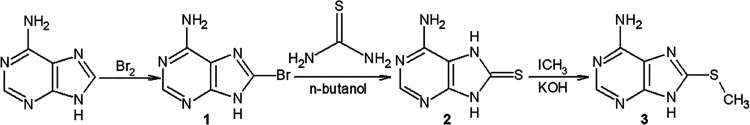
Synthesis of 8-thiomethyladenine.

The compound was prepared from adenine according to a published procedure described by Janeba^22^ in 74% yield (lit. yield 60%) ^1^H NMR δH (500 MHz, DMSO- d6): 13.63 (brs, 1H, NH), 8.11 (s, 1H, CH), 7.47 (brs, 2H, NH_2_) (see Figures 1 and S1).

#### 2.1.2 Synthesis of 6-Amino-7(H)-purine-8(9H)-thione **2**

The compound was obtained via the modified procedure described by Janeba.^23^ To the solution of 8-bromoadenine (2.0 g, 9 mmol) in n-butanol (50 mL) was added thiourea (5.67 g, 70 mmol), and the mixture was refluxed for 28 h. After this time, the obtained precipitate was drained under reduced pressure and recrystallized from water (yield 52%). ^1^H NMR δH (500 MHz, DMSO- d6): 12.97 (brs, 1H, NH), 12.49 (brs, 1H, NH), 8.04 (s, 1H, CH), 7.14 (brs, 2H, NH_2_) (see Figures 1 and S2).

#### 2.1.3 Synthesis of 8-Thiomethyladenine **3**

The compound was obtained via the modified procedure described by Janeba.^23^ To a spherical round bottomed flask were added 6-amino-7*H*-purine-8(9)*H*-thione (250 mg, 1.495 mmol), methyl iodide (236 mg, 1.660 mmol), and 1.5 M aqueous solution of KOH (8.5 mL) and the mixture was stirred in a closed flask for 1 h at 15 °C. Then, the mixture was neutralized with acetic acid, and the obtained precipitate was drained under reduced pressure and purified using a Pure Chromatography System (Flash) with 60% yield. ^1^H NMR δH (500 MHz, DMSO-d6): 12.99 (brs., 1H, NH), 8.03 (s, 1H, CH), 7.00 (brs, 2H, NH_2_), 2.67 (s, 3H, CH_3_) (see Figures 1 and S3). HRMS, theoretical mass [M-1]^−^ = 180.0349 Da; experimental mass [M-1]^−^ = 180.0366 Da (see Figure S4).

### 2.2 Crossed Electron-Molecule Beam Experiment

DEA to ASCH_3_ in the gas phase was studied in a CEMB experiment, which was described in detail in ref (24). The effusive beam of ASCH_3_ molecules in the gas phase was produced by sublimation of the solid sample in a resistively heated copper oven inside of the vacuum chamber. The experiments were performed at chamber pressure and sample temperature of about 5 × 10^–8^ mbar and 367 K, respectively. The sample vapor entered the interaction region with the electron beam through a capillary of 1 mm diameter. The monochromatized electron beam was produced by a hemispherical electron monochromator. In the present experiment, the energy resolution of the electron beam was about 130 meV at full width half-maximum. The electron current was about 35 nA and was monitored with a picoamperemeter. The anions formed upon electron attachment were extracted from the interaction region by a weak electrostatic field into a quadrupole mass filter where they were mass-selected and subsequently detected by a channeltron-type secondary electron multiplier in single pulse counting mode. Before the measurements of negative ions, the temperature dependence of the electron ionization mass spectrum at the electron energy of 70 eV was measured. This measurement aimed to ensure that no significant thermal decomposition occurred until the sublimation temperature was chosen for the negative ion measurements. The electron energy scale and energy resolution were determined by measuring the well-known ion yield for the formation Cl^–^/CCl_4_ at 0 eV.^25^

### 2.3 Quantum Chemical Calculations

DEA profiles and proton affinities were calculated using the fully optimized geometries of the reactants at the MPW1K level,^26^ employing the 6-31++G(d,p) basis set.^27,28^ The polarization continuum model (PCM) was used to mimic the solvent.^29^ All of the optimized geometries were found to be geometrically stable, as verified by harmonic frequency analysis (all force constants were positive for minima, while only one of them was negative for the first-order transition states). Additionally, the intrinsic reaction coordinate procedure^30^ was used to ensure that the obtained transition state connects the proper minima. The Gibbs free energies of particular elementary reactions (Δ*G*s) and activation free energies (Δ*G*s*) were estimated as Δ*E*s (electronic energy change) between the product and substrate (or between the substrate and transition state for activation energy) corrected for zero-point vibration terms, thermal contributions to energy, the pV term, and the entropy term. These terms were calculated in the rigid rotor-harmonic oscillator approximation for *T* = 298 K and *p* = 1 atm.^31^

For the interpretation of CEMB experiments, unconstrained geometry optimizations of the species involved in the fragmentation reactions were carried out at the M06-2X^32^ level using the aug-cc-pVTZ^33^ basis set. The electronic energies (E) corrected for the zero point energy (ZPE) were employed in calculations of thermodynamic thresholds. The energy difference, Δ*E*(ZPE), serves as the determinant for the thermodynamic threshold in cases where no transition state exists during the substrate-to-product transformation such as homolytic bond dissociation. However, if a transition state is present, its electronic energy surpasses that of the substrate. Therefore, even for an exothermic DEA process (negative Δ*E*(ZPE)), the excess electron energy that induces the DEA reaction may exceed zero eV. Similarly, for endothermic processes, the presence of a transition state increases the threshold beyond Δ*E*(ZPE). Consequently, to induce an electron-attachment-initiated reaction with a bottleneck step involving a transition state, the excess electron must possess kinetic energy equal to the difference between the energy of the substrate and that of the transition state. Identifying and characterizing transition states becomes crucial for accurately estimating experimental thresholds.^24^

All the calculations were performed with the Gaussian 16 suite of programs.^34^

### 2.4 Stationary Radiolysis of 8-Thiomethyladenine

#### 2.4.1 Water Solution

Radiolysis of a 10^–4^ M solution of 8-thiomethyladenine was performed in Eppendorf probes, in the presence of 0.03 M *tert*-BuOH as an ^•^OH free radical scavenger and phosphorus buffer (10 mM, pH 7) using CellRad X-ray Cabinet (Faxitron X-ray Corporation). The voltage and current of the X-ray tube were equal to 130 kV and 0.1 mA, respectively. A 0.5 mm Al filter was used. The samples were deoxygenated by purging with argon for 3 min and exposed to 300 Gy (5.83 Gy min^–1^) of radiation dose. The radiation-chemical yield of solvated electrons in water, *G*(*e*_solv_) is equal to 2.8 × 10^–7^ mol/J.^35^

#### 2.4.2 Acetonitrile

Radiolysis of a 10^–4^ M solution of 8-thiomethyladenine in dry ACN was performed in small flasks, using a CellRad X-ray Cabinet (Faxitron X-ray Corporation). The tube voltage, current, and filter were set as for radiolysis in water. The samples were deoxygenated by purging with argon for 3 min and exposed to 300 Gy (5.83 Gy min^–1^) of radiation dose. The radiation-chemical yield of solvated electrons in ACN, *G*(*e*_solv_) is equal to 1.6 × 10^–7^ mol/J.^36^ Then, each of the samples was evaporated under a vacuum, and the obtained residue was dissolved in the same amount of water.

### 2.5 Potentiometric Titration of 8-Thiomethyladenine Solution

Potentiometric titrations were performed at 298.15 K, using a new Cerko Lab System EQSOL software based on an algorithm presented by Kostrowicki and Liwo,^37,38^ fitted with a 5 mL Hamilton’s syringe, a pH combined electrode (Hydromet ERH-13-6) calibrated according to IUPAC recommendations,^39^ and a self-made measuring cell (30 mL) equipped with a magnetic stirrer. The temperature was controlled by using a Lauda E100 circulation thermostat. The composition of the titrant solution is as follows: 1 mM 8-thiomethyladenine and 5 mM HClO_4_. The solutions were potentiometrically titrated with a standardized 24 mM NaOH solution in the pH range from 3 to 11. The experiment consisted of injecting 0.02 mL of the titrant at 2 min intervals into the reaction cell, which initially contained 5.0 mL of the titrant solution. The dissociation constants were refined by least-squares calculations using the Hyperquad2008 (ver. 5.2.19) computer program.

## 3 Results and Discussion

### 3.1 CEMB Experiments for ASCH_3_

**Figure 2:**
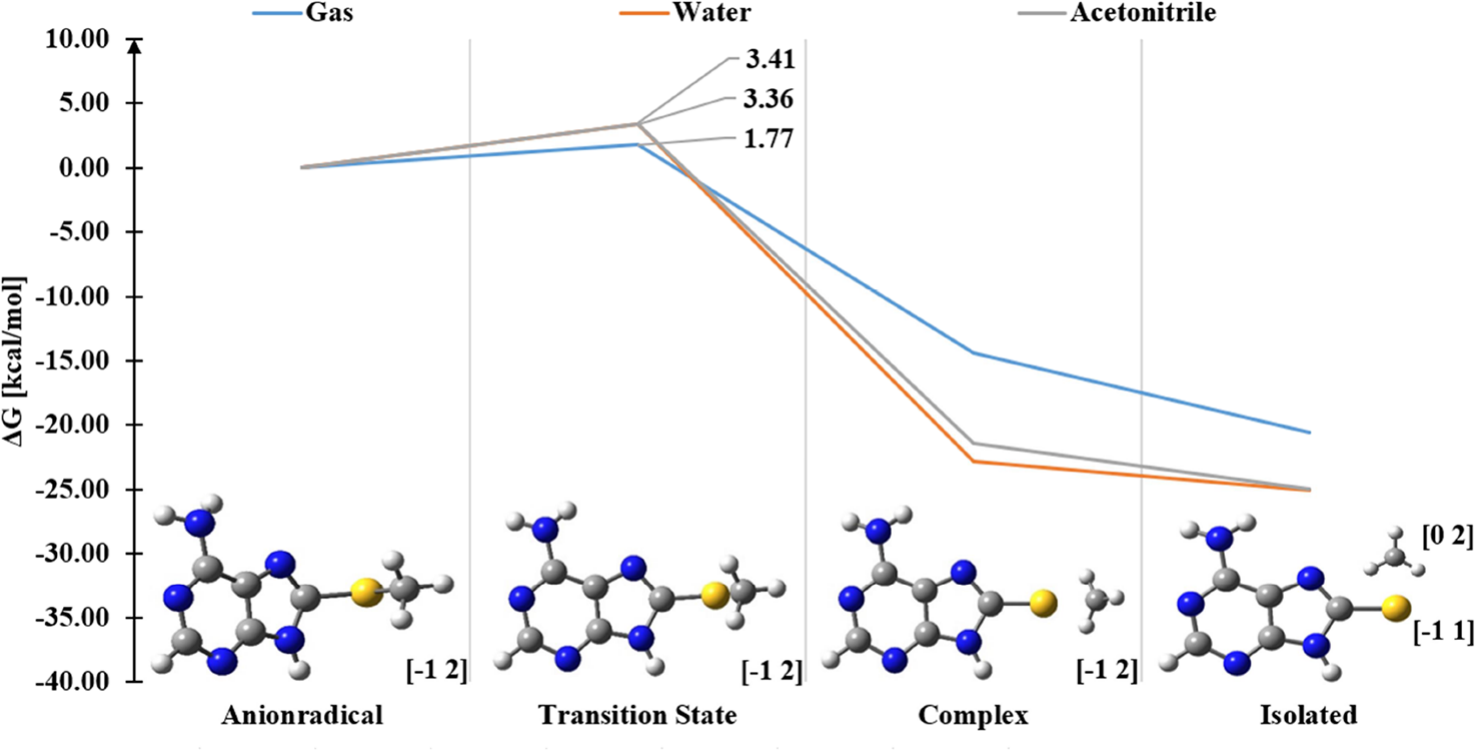
DEA (in the Gibbs free energy scale) profiles of the ASCH_3_ anion radical under three different conditions. Marked as blue: gas phase, orange: water (PCM), and gray: acetonitrile (PCM). The charge and multiplicity of particular species are shown in the square brackets.

The DEA profile for ASCH_3_ calculated at the MPW1*K*/6-31++G(d,p)/PCM level is depicted in Figure 2. The kinetic barrier of 1.8 kcal/mol and the thermodynamic stimulus of −20.6 kcal/mol suggest an efficient DEA process in the gas phase. These computational findings were confirmed using CEMB experiments. Briefly, a molecular beam of ASCH_3_ was crossed with a beam of electrons of well-defined energy, and the molecular fragment anions were analyzed with a mass spectrometer to study the fragmentation yield versus the incident electron energy in the electron energy range from ∼0 to 14 eV.

**Figure 3:**
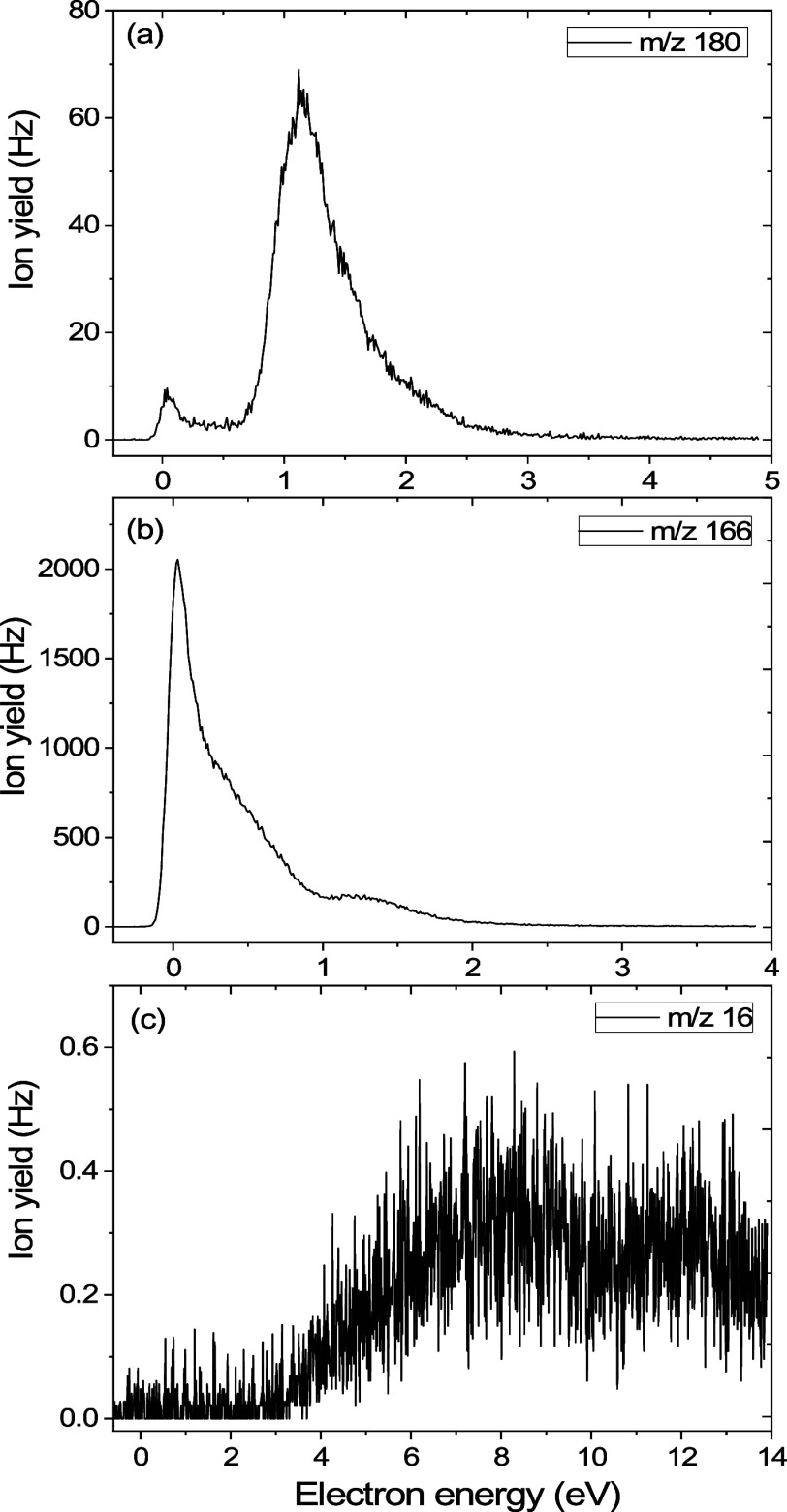
Anion efficiency curves of the fragment anions formed upon electron attachment to ASCH_3_, (a) ASCH_2_^–^ (*m*/*z* 180), (b) AS^–^ (*m*/*z* 166), and (c) NH_2_^–^ (*m*/*z* 16).

The most intense CEMB signal originates from the AS^–^ anion (Figure 3b). Two other fragment anions ((ASCH_2_)^−^ and (NH_2_)^−^), however of much lower intensity, were recorded in the CEMB experiment (see the anion efficiency curves depicted in Figure 3a,c).

The experimental thresholds for each anion fragment are listed in Table 1 and are compared with the computationally obtained values. The parent anion [mass-to-charge ratio (*m*/*z*) equal to 181] was not observable within the detection limit of the apparatus.

**Table 1:** Summary of the Observed Fragment Anions in Terms of Masses, Structural Assignments, and Their Corresponding Maxima on the Anion Efficiency Curves, as well as the Experimental and Calculated Thresholds (Δ*E*_0_)

mass (*m*/*z*)	anion	peak positions (eV)			*T* (K)	threshold (eV)	
						exp.	theory
180	(ASCH_2_)^−^	1.1	1.4	1.6	367	0.7	0.81^a^
							1.71^b^
							2.71^c^
							3.64^d^
166	(AS)^−^	≈0	0.3	1.2	367	≈0	–0.17
16	(NH_2_)^−^	7.7	12.0		365	4.0	4.11

a Reaction 1a.

b Reaction 1b.

c Reaction 1c.

d Reaction 1d (see Figure 5).

**Figure 4:**
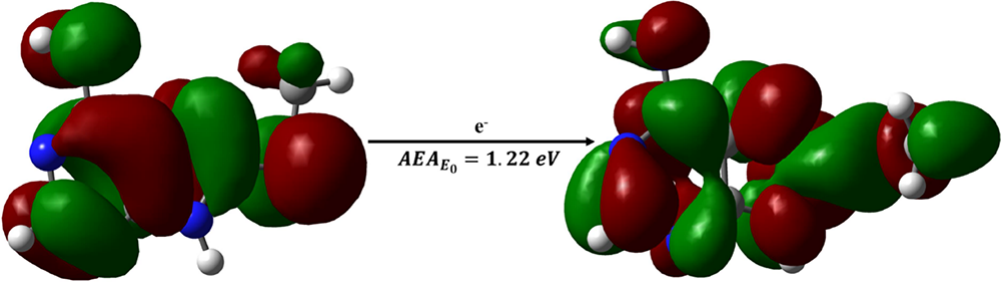
Adiabatic electron affinity (AEA_E0_) in terms of the zero-point energy-corrected difference between the electronic energies of the neutral and anion radical calculated at the M06-2X/aug-cc-pVTZ level. The following color codes were used to indicate particular atoms: white for H, gray for C, blue for N, and yellow for S. A LUMO/SOMO orbital was superimposed on the structures of the ASCH_3_ neutral form (left) and the anion radical (right).

The adiabatic electron affinity of ASCH_3_ was computed to be approximately 1.22 eV (see Figure 4) at the M06-2X/aug-cc-pVTZ level which is close to the energy of transition state for the AS^–^ release from the ASCH_3_ radical anion (see Figure 6). The lacking energy of 0.22 eV (the difference between 1.22 eV and the energy of TS for the AS^–^ release (see Figure 6, TS_166 structure)) can originate from vibrational excitation of ASCH_3_ molecules due to the heating process in the molecular beam oven.^40^ This situation indicates that the energy released due to the electron attachment process makes overcoming the kinetic barrier probable and, in consequence, may lead to a dramatic shortening of the lifetime of the ASCH_3_ anion which justifies the lack of the ASCH_3_^•–^ signal in the CEMB experiment.

**Figure 5:**
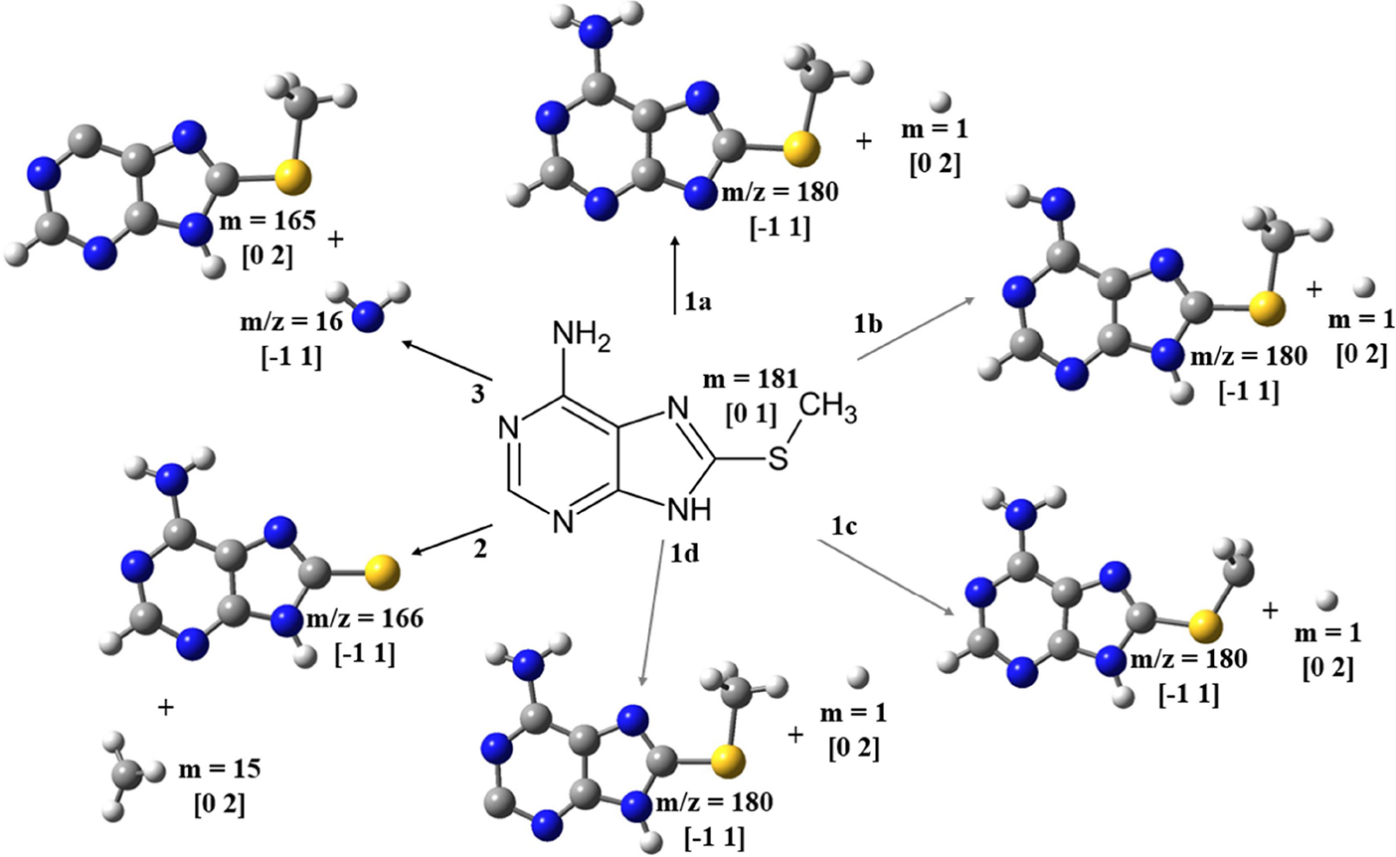
Dissociation pathways in the ASCH_3_ molecule upon low-energy electron attachment. The experimentally detected fragments are denoted with a mass-to-charge ratio of 180 (1a-d), 166 (2), and 16 (3). The charge and multiplicity are shown in parentheses for each structure. The following color codes were used to indicate particular atoms: white for H, gray for C, blue for N, and yellow for S.

Electron attachment to ASCH_3_ leads to the formation of three fragment anions, which have been presented in Figure 5 illustrating the structures of possible anion radicals/neutrals following the dissociation process.

The heaviest anion at *m*/*z* 180 (Figure 3a) was recorded upon the cleavage of the C–H or N–H bond, leading to the formation of the ASCH_2_^–^ anion and the H radical (eq 1):
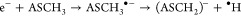
1

**Figure 6:**
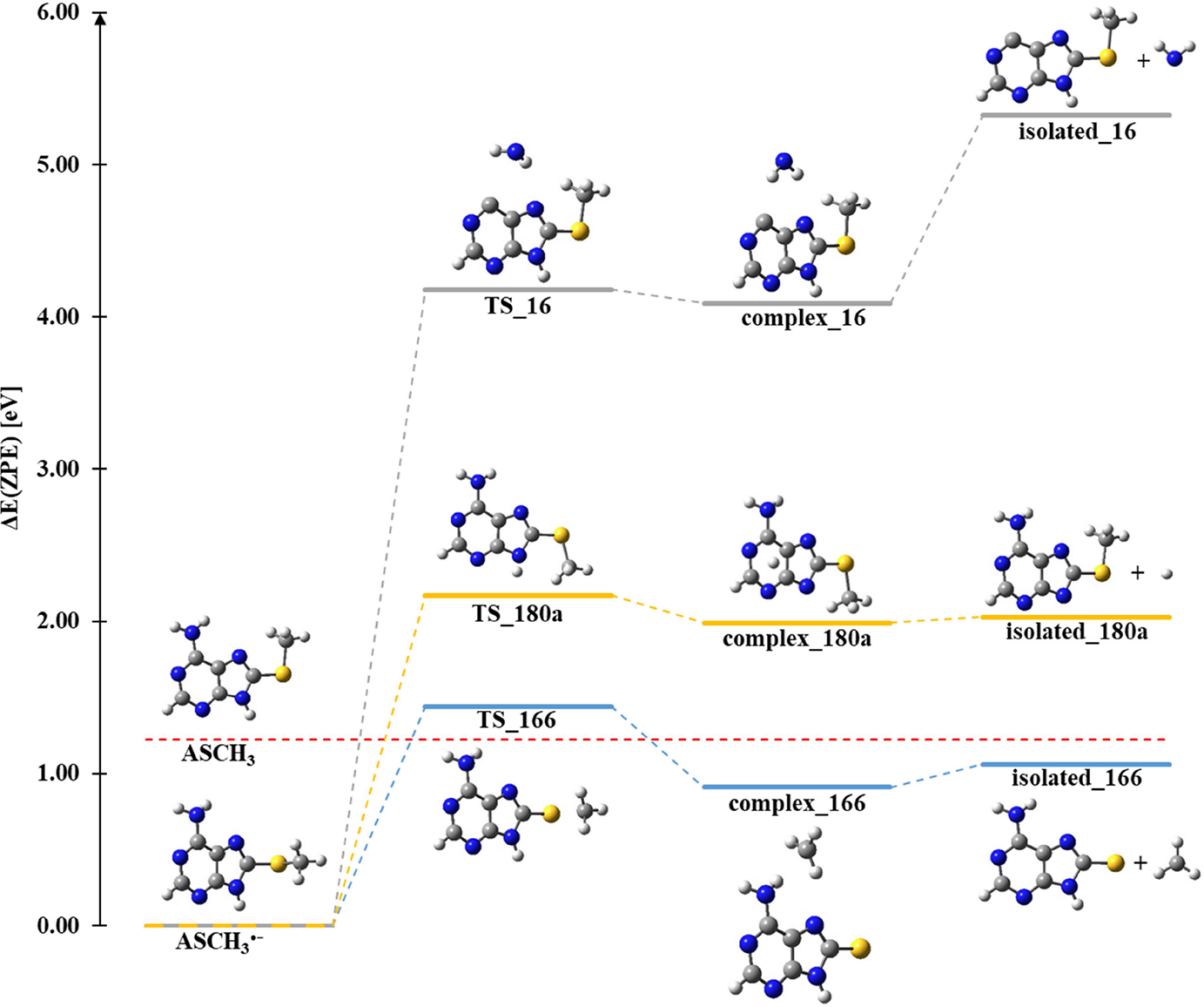
Single bond cleavage pathways, leading from ASCH_3_ anion radical to the most abundant anionic products at *m*/*z* 16, *m*/*z* 166, and *m*/*z* 180(a). TS_x, complex_*x*, and isolated_*x,* where *x* refers to the value of *m*/*z*, stand for the transition state, product complex, and isolated monomers, respectively.

The reaction energy for the release of ASCH_2_^–^ spans a range from 0.81 to 3.64 eV depending on the site of C–H/N−H rupture (Table 1). The activation energy of the most favorable reaction (thermodynamic stimulus of 0.81 eV) amounts to 2.17 eV (Figure 6, TS_180a structure). The difference between this activation energy and AEA is equal to 0.95 eV and is near the threshold energy of the main peak (0.7 eV) in the anion efficiency curve for ASCH_2_^–^ formation. The minor first peak in the ion yield at about zero eV can be ascribed to an impurity.

The second heaviest fragment anion, yet forming with the highest yield (that significantly exceeds the second most abundant anion in intensity, see Figure 3b), is observed at *m*/*z* 166 following DEA to ASCH_3_. This anion is generated through the cleavage of the S-CH_3_ bond, as illustrated in Figure 3b and described by eq 2:
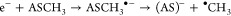
2

The formation of this anion is favorable (thermodynamic threshold of −0.17 eV, see Table 1) and has a low activation barrier of 0.22 eV (see Figure 6 TS_166). As pointed out above, the first peak in the anion efficiency curve of (AS)^−^ can be ascribed to vibrationally excited molecules before the electron attachment event. In this case, sufficient internal energy is present in the neutral molecule to overcome the barrier after the attachment of an ∼zero eV electron. The (AS)^−^ ion yield shown in Figure 3b also indicates an abundant shoulder in the tail of the zero eV peak. Multi-Gaussian peak fit analysis of the ion yield reveals an underlying peak near 0.3 eV. In contrast to the zero eV peak, this ion signal can be associated with electron attachment to neutral molecules in the vibrational ground state. In this case, the initial kinetic energy of the electron is deposited as the excitation energy in the ASCH_3_^•–^ transient negative ion and leads to the decay over the barrier.

The lightest anion formed upon DEA to ASCH_3_ was registered at *m*/*z* equal to 16 (see Figure 5, reaction 3). This species results from the C–N bond cleavage between the C6 atom of the purine ring and the −NH_2_ group and may be written as follows (eq 3):
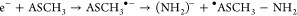
3

This reaction involves 2.96 eV of energy (activation barrier, see TS_16 in Figure 6) and the threshold is equal to 4.11 eV (from the neutral form of the ASCH_3_). This finding agrees well with the experimentally determined threshold, which was found at 4.0 eV (see Table 1 and Figure 3c). Above this threshold, the (NH_2_)^−^ anion is formed over a wide energy range up to the maximum energy measured (14 eV) but its overall intensity is very low compared with the other two fragment anions detected.

The results of the experimental studies in the gas phase and quantum chemical calculations stand in good agreement. A significant prevalence of an anion with a mass-to-charge ratio of 166 observed in the CEMB experiments may suggest that it also occurs as the primary species during the DEA process in the aqueous phase.

### 3.2 Radiolysis of ASCH_3_ in Water or Acetonitrile

**Figure 7:**
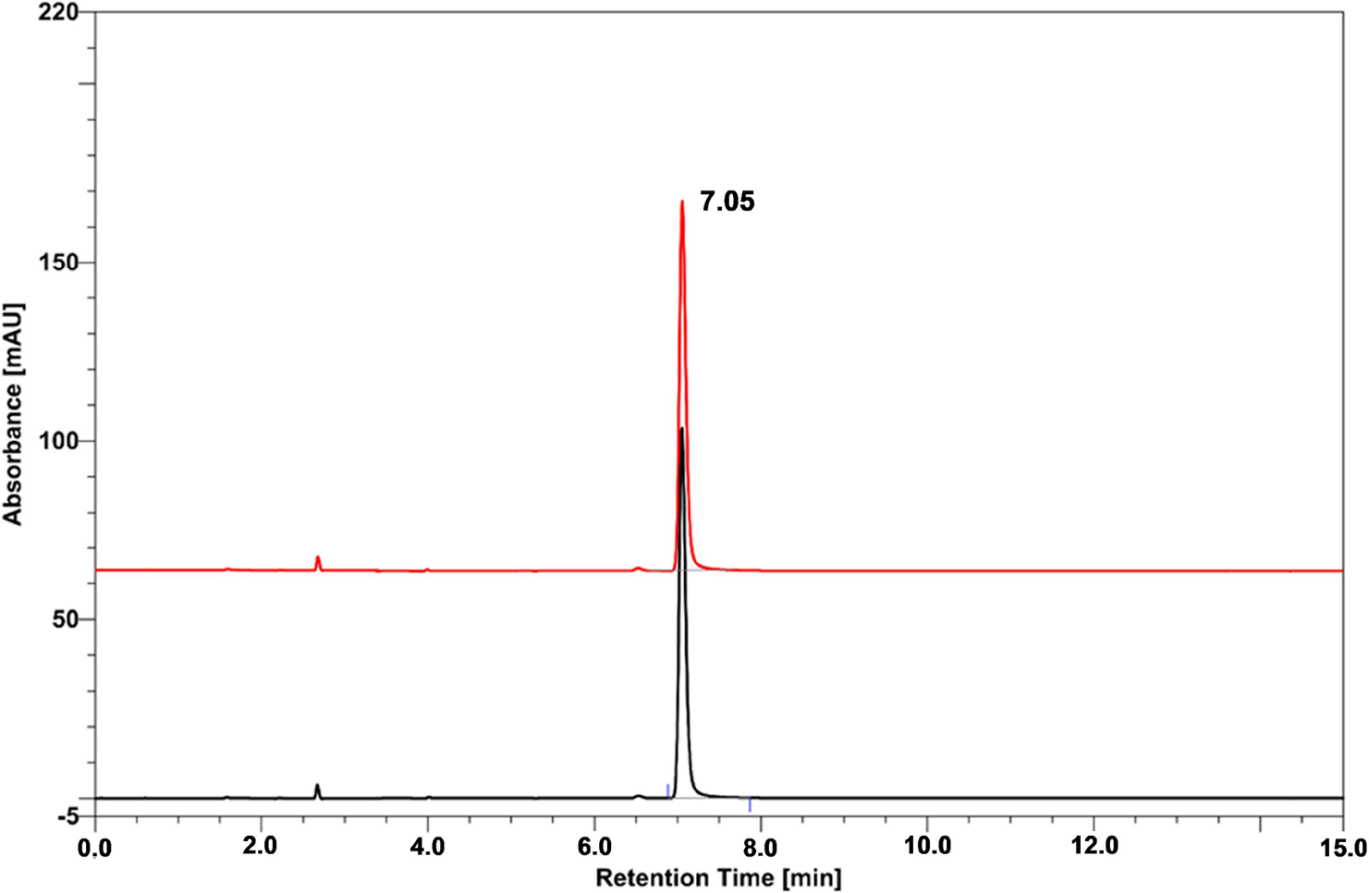
HPLC analysis of ASCH_3_ in water before (black line) and after irradiation with a dose equal to 300 Gy (red line).

An effective radiosensitizer for cancer hypoxic cells has to act in an aqueous solution, i.e., in a physiological environment.^41^ Therefore, to demonstrate that solvated electrons trigger electron attachment-induced chemistry, radiolysis of an aqueous ASCH_3_ solution was conducted under reducing conditions. The experiment was performed in the presence of tertbutyl alcohol (*tert*-BuOH) as the ^•^OH radicals scavenger and phosphorus buffer (10 mM, pH 7) to keep the pH of the solution at the physiological level. Before irradiation with a dose of 300 Gy (5.83 Gy min^–1^), the samples were deoxygenated by purging with argon to mimic the hypoxic conditions characteristic of solid tumors. As depicted in Figure 7, the result of the above-described radiolytic experiment proved to be quite surprising (taking into account the outcome of CEMB experiments combined with the QM calculations). Namely, the stationary radiolysis of the aqueous ASCH_3_ solution does not lead to its degradation, although the DEA profile obtained at the MPW1*K*/6-31++G(d,p)/PCM method indicates that the DEA process should proceed as the kinetic barrier of the dissociation process amounts to only 3.4 kcal/mol (Figure 2).

A hypothesis that might explain the findings described above could be a protonation of the primary radical anion formed due to electron attachment to the ASCH_3_ molecule. Indeed, such a protonation enlarges the barrier for electron transfer from the SOMO π orbital of the primary anion to the respective σ* orbital, i.e., for the process that is responsible for electron attachment-induced dissociation. Protonation of radical anions was invoked in the past to explain the hindering of the DEA process in native nucleotides^42^ or halo-thiouridines.^43^

**Figure 8:**
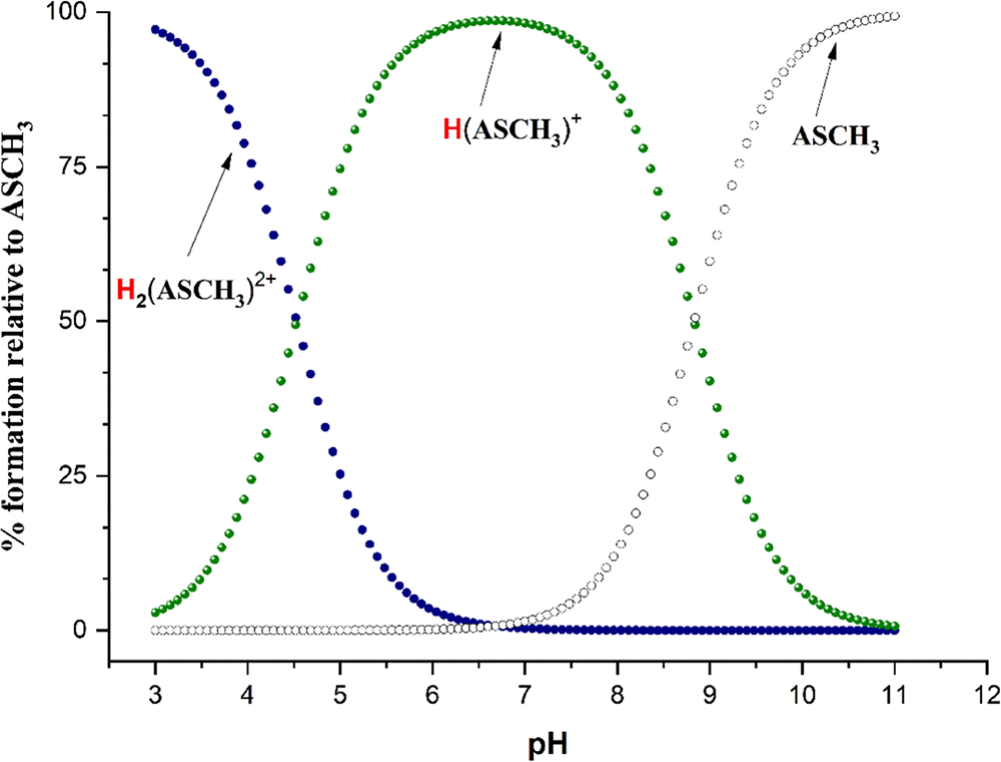
Species distributions of ASCH_3_ as a function of pH.

To determine the protonation state of ASCH_3_ under the experimental conditions, potentiometric measurements were conducted. The equilibrium model presented in Table 2 has given the best fitting of calculated data to the potentiometric titration ones. The results indicate that ASCH_3_ exists as the monoprotonated species at physiological pH (see Figure 8), with the proton most likely linked at the N1 position, as indicated by the relative theoretical stabilities gathered in Table 3.

**Table 2:** p*K*_a_ Values (Standard Deviation Values in Parentheses) of the Adenine Derivative (ASCH_3_)

no.	equilibrium model	equilibrium expression	p*K*_a_ value
1	H_2_(ASCH_3_)^2+^ = H^+^+ H(ASCH_3_)^+^	*K*_a1_ = [H^+^][H(ASCH_3_)^+^]/ H_2_(ASCH_3)_)^2+^]	4.53 (0.10)
2	H(ASCH_3_)^+^ = H^+^ + (ASCH_3_)	*K*_a2_ = [H^+^][(ASCH_3_)]/[H(ASCH_3_)^+^]	8.83 (0.09)
3	H_2_O = H^+^ + OH^–^	*K*_H2O_ = [H^+^][OH^–^]	14.00 (const.)

**Table 3:** Relative Gibs Free Energy (Δ*G*) for ASCH_3_ Protonated at Chosen Sites^a^

position	Δ*G* [kcal/mol]
N1	0.0
N3	1.9
N7	4.9

a Values were calculated for 298 K and at the MPW1*K*/6-31++G(d,p)/PCM level.

The discrepancy between the low theoretical activation barrier and lack of radiolytic degradation, as well as the protonation of ASCH_3_ at physiological pH, suggests that the formation of the neutral radical ASCH_3_(+H), i.e., electron attachment to protonated ASCH_3_, may inhibit the DEA process.

**Figure 9:**
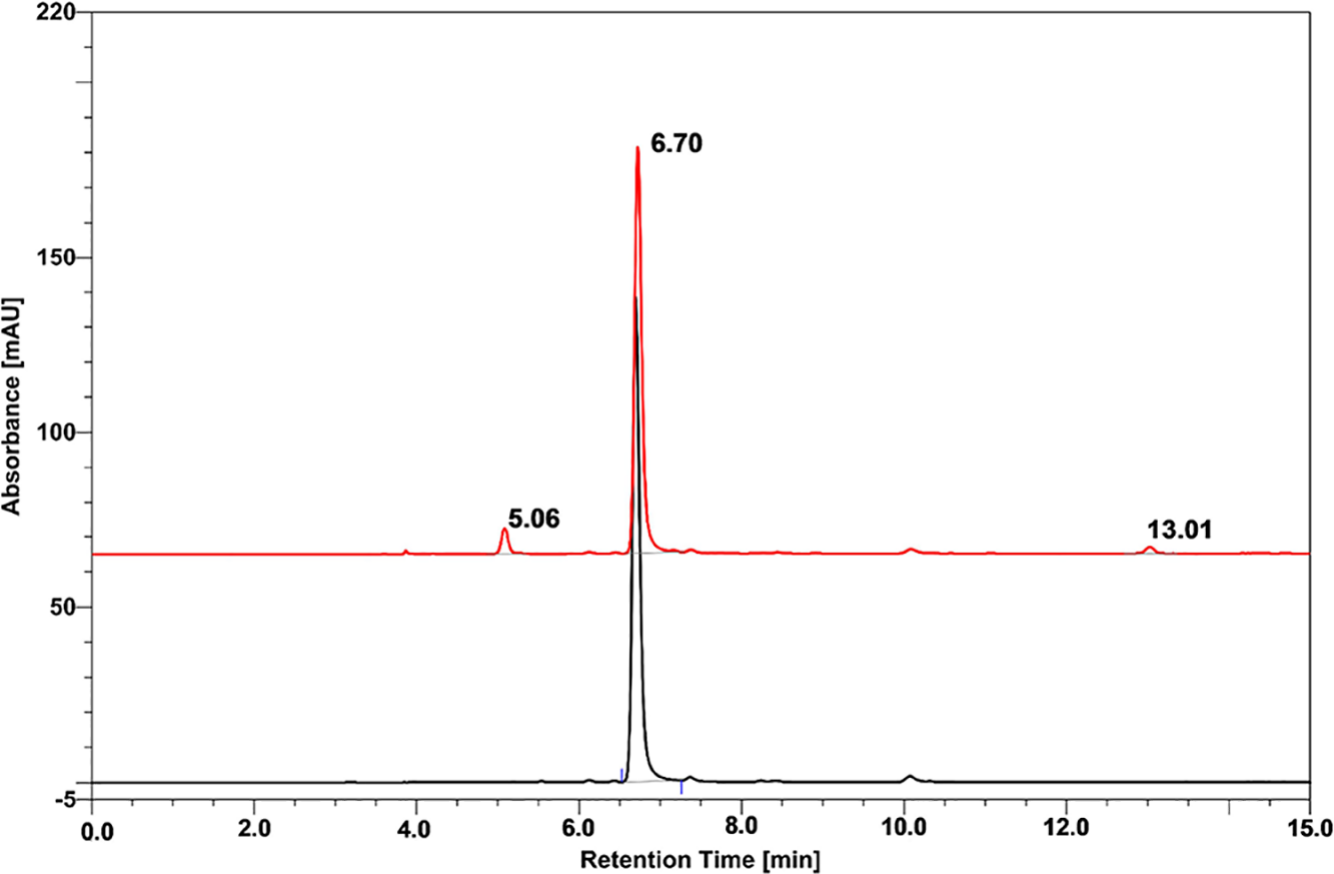
HPLC analysis of ASCH_3_ in ACN before (black line) and after irradiation with a dose equal to 300 Gy (red line).

To confirm this hypothesis, radiolytic studies were also performed in an aprotic solvent, acetonitrile (ACN), where protonation of the neutral form of ASCH_3_ or its anion cannot take place. A solution of ASCH_3_ was prepared in dry ACN, deoxygenated, and irradiated with the same dose as for the aqueous solution. The results of the experimental studies, presented in Figure 9, demonstrate that under these conditions dissociation of the compound induced by electron attachment does take place [see new peaks (retention times equal to 5.06 and 13.01 min) in the chromatogram shown in Figure 9].

The MS/MS spectra in negative ionization mode show that the observed at 5.06 min peak (TIC; MS spectrum) corresponds to the *m*/*z* 196.03 → 181.03 transition may be explained by CH_3_[ASCH_3_]H → ASCH_3_H structural transformation, while the second peak at 13.01 min (TIC–MS spectrum) corresponding to the *m*/*z* 208.03 → 180.06 → 165.04 transition may indicate the H[AS]CH_3_C(H)N^–^ → ASCH_3_ → AS^–^ transformation (see Figures S5–S8 in the Experimental section (e) in the Supporting Information).

The first product with *m*/*z* of 196.03 is related to the attachment of two individuals derived from the stationary radiolysis of acetonitrile, a methyl radical ^•^CH_3_, and hydrogen atom H to the structure of 8-thiomethyladenine. Detachment of the methyl group from the structure gives a mass equal to 181.03 Da. The second product with *m*/*z* equal to 208.03 is probably a product of DEA, where the thiomethyl derivative undergoes electron-induced degradation involving the breakage of the S-CH_3_ bond and addition of CH_3_C(H)N and H^•^ radicals to the intermediate structure (the latter radicals forms in the radiolysis of ACN).^36^ Elimination of the CNH_2_ group leads to the anion of *m*/*z* equal to 180 and the subsequent removal of the CH_3_ group gives the structure characterized by *m*/*z* of 165 anion, which corresponds to the 8-thioadenine anion, as in the experiment performed in the gas phase.

**Figure 10:**
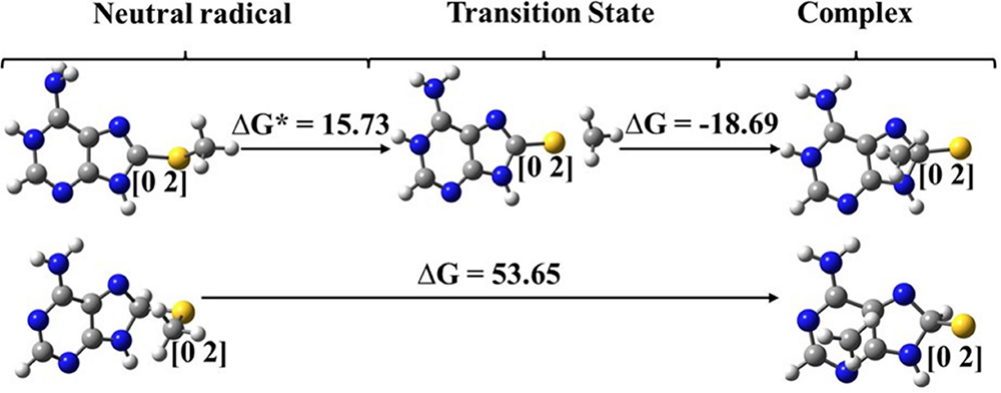
Gibbs free energy profiles for the dissociation of the ASCH_3_ radical anion protonated at N1 (top panel) and N2 (bottom panel), calculated for 298 K at the MPW1*K*/6-31++G(d,p)/PCM level.

When considering the DEA process for the radical anion of ASCH_3_ in an aqueous solution, the activation barrier at the MPW1*K*/6-31++G(d,p) level is only 3.4 kcal/mol (ΔG*, see Figure 2). In the case of the radical anion protonated at N1, the barrier increases to 15.7 kcal/mol (see the upper panel of Figure 10). In this scenario, when the radical anion is protonated at the N1 position, one might still expect products of the radiolysis of the aqueous ASCH_3_ solution since with the kinetic barrier of only 15.7 kcal/mol, and according to transition state theory, the half lifetime of the ASCH_3_(+H) radical protonated at N1 amounts to c. 0.04 s at 298 K. However, as indicated by Figure 7 this is not the case (no radiolysis products are observed). Hence, one can explain the lack of radiolytic reactivity of ASCH_3_ either by referring to competitive processes like (i) hydrogen atom transfer between ASCH_3_(+H)^•^ and the *tert*-BuOH radical (no kinetic barrier, Δ*G*_r_ = −82.8 kcal/mol at the MPW1*K*/6-31++G(d,p)/PCM level); the latter species forms in a significant amount during scavenging the OH^•^ radicals or (ii) the N1 → C8 tautomerization of the ASCH_3_(+H)^•^ radical. As far as the latter process is concerned, one should note that ASCH_3_(+H)^•^ protonated at C8 is by as much as 13.6 kcal/mol more stable than that protonated at N1 (Table 4). In water proton transfer (PT) between N1 and C8 sites can be realized by a sequence of proton hops between hydrogen-bonded water molecules. A bottleneck PT is the first reaction in the sequence, i.e., a transfer of proton between ASCH_3_(+H)^•^ and the neighbor water molecule (ca. 18.3 kcal/mol at the MPW1*K*/6-31++G(d,p)/PCM level), leading to the formation of the radical anion and H_3_O^+^ cation. The AIMD simulations indicate that the formation of the H_3_O^+^/OH^–^ anion pair from two water molecules requires a 23.8 kcal/mol^44^ barrier to be overcome in a water solution. Assuming, thus, 18.3 kcal/mol (see above) as a limiting value for the kinetic barrier of PT in the ASCH_3_ system, one can calculate that the equilibrium concentration of ASCH_3_(+H)^•^ protonated at C8 will be attained after ca. 0.36 s [assuming pseudo-first-order kinetics for PT (the concentration of water in the water solution is constant and amounts to ca. 55.5 kcal/mol), the activation barrier of 18.3 kcal/mol, and 99% degree of conversion]. As depicted in Figure 10, the barrier of S-CH_3_ bond dissociation increases from 15.3 kcal/mol in ASCH_3_(+H)^•^ protonated at N1 to 53.7 kcal/mol in ASCH_3_(H)^•^ protonated at C8. The latter value shows that the S-CH_3_ dissociation is completely hindered at 298 K for the ASCH_3_(+H)^•^ radical protonated at C8. It seems, therefore, that both processes (i) and (ii) may explain the lack of radiolytic reactivity of ASCH_3_ in an aqueous solution.

**Table 4:** Relative Gibbs Free Energy of Tautomerization (Δ*G*_t_) of the ASCH_3_(+H)^•^ Radical for 298 K, Calculated at the MPW1*K*/6-31++G(d,p)/PCM Level

site of protonation	Δ*G*_t_ [kcal/mol]
N1	13.6
N3	11.8
N7	8.7
C2	6.9
C6	19.1
C8	0.0
NH_2_ group	28.6

## 4 Conclusions

The findings presented in this paper lead to the conclusion that effective radiosensitization using derivatives of nucleic bases is not solely determined by the molecular structure of the potential sensitizer that ensures the low stability of its radical anion and facilitates dissociation, as previously assumed. The protonation of the radical anion, especially involving proton-acceptor centers responsible for localizing the excess electron, leads to a significant increase in the barriers of the DEA process. In extreme cases, this completely inhibits the dissociation of the compound, depriving it of radiosensitizing properties. The same effect (protonation of radical anions) may be responsible for inhibiting the formation of single-strand breaks in aqueous solutions of native DNA, a damage type induced in DNA by electrons under anhydrous conditions. In the future design of new radiosensitizers, it seems necessary to consider not only the molecule’s affinity to an electron and the ease of the DEA process but also their potential for protonation in an aqueous solution.

## Supporting Information Available

XYZ geometries of all stationary points, details of LC/MS measurements, and ^1^H NMR and MS spectra (PDF)
Raw data originating from the CEMB experiments (XLSX)
