# Supplementary material for: Surprising Radiolytic Stability of 8-Thiomethyladenine in an Aqueous Solution
Source: J Phys Chem B. 2024 Apr 5;128(15):3621–30. doi: 10.1021/acs.jpcb.4c01033 (PMC11033863; doi:10.1021/acs.jpcb.4c01033)
Supplement: Supplementary file 1 — jp4c01033_si_001.pdf [file jp4c01033_si_001.pdf]

Supporting Information

Surprising Radiolytic Stability of 8-Thiomethyladenine in an Aqueous Solution

Magdalena Datta,[1,#] Adrian Szczyrba,[1,#] Magdalena Zdrowowicz,[1] Dariusz Wyrzykowski,[1] Olga Ciupak,[2] Sebastian Demkowicz,[2] Farhad Izadi,[3] Stephan Denifl,[3*] Janusz Rak[1*]

[1] *Faculty of Chemistry, University of Gdańsk, Wita Stwosza 63, Gdańsk 80-308, Poland*

[2] *Department of Organic Chemistry, Faculty of Chemistry, Gdańsk University of Technology, Narutowicza 11/12, 80-233 Gdańsk, Poland*

[3] *Institut für Ionenphysik und Angewandte Physik and Center for Biomolecular Sciences Innsbruck, Universität Innsbruck, Technikerstrasse 25, A-6020 Innsbruck, Austria*

* Authors to whom correspondence should be addressed.

Electronic mails: janusz.rak@ug.edu.pl (J. Rak), stephan.denifl@uibk.ac.at (S. Denifl)

# These authors contributed equally.

XYZ of stationary points

a) DEA profiles

For the names of XYZ stationary geometries see Figure 2 in the main text. The solvent is indicated in brackets.

Anionradical (ACN)

Total Electronic Energy: -904.780553154

C	-2.032848	0.880117	0.051513
C	-0.745297	0.385708	-0.174690
C	-0.626912	-1.016664	-0.124235
N	-1.608172	-1.876593	0.116627
C	-2.788588	-1.268768	0.318729
N	-3.041239	0.026445	0.297901
N	0.671677	-1.266914	-0.382529
C	1.313097	-0.038777	-0.704728
N	0.428922	0.983268	-0.469856
S	3.024533	0.117254	-0.419698
C	3.234404	0.276367	1.393626
N	-2.296248	2.236985	0.082954
H	4.295145	0.421610	1.584684
H	2.900231	-0.623407	1.902142
H	2.682733	1.136261	1.761560
H	1.062190	-2.173610	-0.550688
H	-3.630310	-1.919488	0.513808
H	-3.255341	2.464673	-0.112390
H	-1.654895	2.787658	-0.460917

TS (ACN)

Total Electronic Energy: -904.772411582

C	-2.104938	0.887866	0.072123
C	-0.816580	0.388296	-0.115838
C	-0.700034	-1.005272	-0.077153
N	-1.687967	-1.869731	0.121935
C	-2.864088	-1.265378	0.287320
N	-3.119270	0.034272	0.272151
N	0.612872	-1.246997	-0.289241
C	1.262682	-0.021211	-0.494164
N	0.381820	0.988903	-0.350405
S	2.946096	0.091195	-0.599440
C	3.856363	0.262199	1.343011
N	-2.365321	2.229707	0.107305
H	4.911731	0.351607	1.106274
H	3.622367	-0.644050	1.888885
H	3.449802	1.156255	1.800290
H	1.047853	-2.145370	-0.370034
H	-3.714255	-1.913773	0.449860
H	-3.320229	2.486959	-0.062021
H	-1.690179	2.817176	-0.346239

Complex (ACN)

Total Electronic Energy: -904.799635380

C	-1.823853	0.395743	-0.759480
C	-0.518043	-0.010356	-0.480596
C	-0.378141	-0.952819	0.529893
N	-1.360670	-1.495763	1.244020
C	-2.547955	-1.032539	0.883914
N	-2.830350	-0.137874	-0.057734
N	0.958958	-1.174513	0.603307
C	1.584391	-0.382258	-0.341501
N	0.698957	0.335057	-1.010548
S	3.289311	-0.403791	-0.535211
N	-2.108487	1.335823	-1.688180
C	0.932285	2.951838	1.959577
H	0.927500	2.291632	1.108181
H	0.027711	3.454488	2.257976
H	1.841552	3.110347	2.514498
H	1.434055	-1.800958	1.224430
H	-3.398511	-1.431167	1.419863
H	-3.060838	1.414619	-1.989021
H	-1.401408	1.564927	-2.359457

Anion (ACN)

Total Electronic Energy: -864.962766288

C	-1.747049	0.855040	-0.005524
C	-0.428116	0.397984	-0.006636
C	-0.263605	-0.980917	-0.001206
N	-1.233745	-1.891502	0.002723
C	-2.434967	-1.334207	0.003904
N	-2.740947	-0.040513	0.001290
N	1.081779	-1.161633	0.001477
C	1.687423	0.080770	-0.001118
N	0.782241	1.043030	-0.006418
S	3.397015	0.240252	0.002702
N	-2.059108	2.169298	-0.048301
H	1.573535	-2.034659	0.006336
H	-3.276507	-2.013398	0.009228
H	-3.000653	2.428886	0.175002
H	-1.342277	2.832360	0.174289

Radical (ACN)

Total Electronic Energy: -39.8364589997

H	-0.932545	-0.538242	-0.000612
H	0.932415	-0.538467	-0.000612
H	0.000130	1.076726	-0.000612
C	0.000000	-0.000003	0.000306

Anionradical (H_2O)

Total Electronic Energy: -904.782123897

C	-2.033506	0.879889	0.051615
C	-0.745958	0.385926	-0.174939
C	-0.626797	-1.016354	-0.125058
N	-1.607832	-1.876644	0.115854
C	-2.788342	-1.269520	0.318758
N	-3.041405	0.025606	0.298504
N	0.671763	-1.266153	-0.383514
C	1.312989	-0.037871	-0.704844
N	0.428367	0.984032	-0.469507
S	3.024608	0.118328	-0.419634
C	3.236141	0.274383	1.393768
N	-2.297355	2.236514	0.083976
H	4.298021	0.411747	1.583807
H	2.896206	-0.623878	1.900959
H	2.691189	1.137812	1.763409
H	1.063047	-2.172774	-0.550629
H	-3.629706	-1.920599	0.514126
H	-3.256845	2.464312	-0.109516
H	-1.657570	2.787930	-0.461003

TS (H_2O)

Total Electronic Energy: -904.773927071

C	-2.105259	0.887826	0.072225
C	-0.816926	0.388377	-0.116258
C	-0.700051	-1.005109	-0.077950
N	-1.687751	-1.869784	0.121509
C	-2.863857	-1.265748	0.287881
N	-3.119207	0.033868	0.273046
N	0.612823	-1.246818	-0.290418
C	1.262525	-0.021096	-0.495007
N	0.381605	0.989172	-0.350717
S	2.946159	0.092035	-0.598825
C	3.857418	0.261097	1.343207
N	-2.365987	2.229417	0.107739
H	4.911972	0.356894	1.105455
H	3.628862	-0.648262	1.886172
H	3.446889	1.151417	1.804263
H	1.047689	-2.145303	-0.371328
H	-3.713774	-1.914295	0.451018
H	-3.321326	2.486546	-0.059644
H	-1.692345	2.817371	-0.347434

Complex (H_2O)

Total Electronic Energy: -904.801249223

C	1.778553	-0.815167	0.615479
C	0.457145	-0.614366	0.211981
C	0.284607	0.081555	-0.977167
N	1.249607	0.569793	-1.752659
C	2.453608	0.316565	-1.262901
N	2.767082	-0.335711	-0.147488
N	-1.061601	0.133888	-1.146888
C	-1.659066	-0.514887	-0.082664
N	-0.748325	-0.978024	0.754579
S	-3.366617	-0.648230	0.037423
N	2.096422	-1.445541	1.767975
C	-0.256608	4.059957	1.299188
H	0.047598	3.364740	0.535078
H	0.004847	5.101160	1.215412
H	-0.823605	3.712941	2.146397
H	-1.559022	0.563425	-1.903236
H	3.291150	0.682340	-1.841095
H	3.044300	-1.749343	1.882388
H	1.388879	-1.996359	2.214155

Anion (H_2O)

Total Electronic Energy: -864.964447014

C	-1.747049	0.855040	-0.005524
C	-0.428116	0.397985	-0.006636
C	-0.263606	-0.980917	-0.001206
N	-1.233746	-1.891502	0.002723
C	-2.434967	-1.334206	0.003905
N	-2.740946	-0.040514	0.001290
N	1.081780	-1.161633	0.001477
C	1.687424	0.080769	-0.001117
N	0.782242	1.043031	-0.006418
S	3.397015	0.240252	0.002702
N	-2.059108	2.169298	-0.048301
H	1.573535	-2.034659	0.006336
H	-3.276507	-2.013398	0.009228
H	-3.000652	2.428886	0.175002
H	-1.342277	2.832360	0.174289

Radical (H_2O)

Total Electronic Energy: -39.8364767514

H	-0.926907	-0.548109	0.000583
H	0.938144	-0.528645	0.000583
H	-0.011238	1.076776	0.000583
C	0.000000	-0.000004	-0.000291

Anionradical (GAS)

Total Electronic Energy: -904.687452947

C	-1.930236	1.019023	-0.000116
C	-0.717120	0.318479	-0.000274
C	-0.807745	-1.061925	-0.000026
N	-1.920677	-1.788613	0.000200
C	-2.994947	-1.013467	0.000225
N	-3.067460	0.311676	0.000078
N	0.489625	-1.490622	0.000223
C	1.278873	-0.365343	-0.000161
N	0.591319	0.735429	-0.000350
S	3.011343	-0.512449	-0.000235
C	3.441756	1.244872	0.000570
N	-1.987733	2.353241	-0.000372
H	4.526855	1.286651	0.000623
H	3.053448	1.732634	0.890292
H	3.053497	1.733456	-0.888714
H	0.799228	-2.441758	-0.000358
H	-3.947177	-1.527402	0.000296
H	-2.876982	2.813095	0.001139
H	-1.149374	2.904886	0.000711

TS (GAS)

Total Electronic Energy: -904.691857327

N	0.382276	0.988532	0.322983
C	-0.811354	0.386974	0.092492
C	-0.703161	-1.009163	0.053966
N	0.611747	-1.244558	0.256649
C	1.262672	-0.014991	0.455532
N	-1.697268	-1.869005	-0.125911
C	-2.872955	-1.256622	-0.266144
N	-3.125798	0.044216	-0.248713
C	-2.100818	0.885382	-0.076893
N	-2.346835	2.238617	-0.098375
S	2.939730	0.068498	0.625443
C	3.858870	0.278465	-1.330811
H	4.907989	0.422821	-1.090446
H	3.397623	1.153136	-1.772210
H	3.669828	-0.636962	-1.879524
H	1.057994	-2.134851	0.337514
H	-3.730003	-1.902049	-0.407494
H	-3.282810	2.488779	0.158996
H	-1.624676	2.788274	0.330777

Complex (GAS)

Total Electronic Energy: -904.713224581

C	1.664040	0.848368	-0.089038
C	0.496009	0.094265	-0.070158
C	0.674682	-1.284420	0.038540
N	1.837238	-1.922812	0.121894
C	2.865966	-1.087075	0.099163
N	2.849418	0.237597	0.003657
N	-0.586164	-1.777673	0.049427
C	-1.487859	-0.718587	-0.042672
N	-0.826768	0.430569	-0.118284
S	-3.161240	-1.006476	-0.041564
N	1.643442	2.206950	-0.230741
C	-1.693463	3.743476	0.214791
H	-1.685150	4.396699	-0.643293
H	-1.827988	4.175160	1.193761
H	-1.661525	2.668550	0.088412
H	-0.870795	-2.733165	0.121532
H	3.847158	-1.538437	0.170146
H	2.471829	2.679162	0.074294
H	0.769900	2.657063	-0.025239

Anion (GAS)

Total Electronic Energy: -864.872827417

C	-1.741982	0.844334	-0.014932
C	-0.426240	0.398517	-0.024169
C	-0.262681	-0.986713	-0.013493
N	-1.236227	-1.891228	0.001798
C	-2.437722	-1.330779	0.016273
N	-2.744456	-0.037843	0.012149
N	1.080021	-1.154625	-0.008170
C	1.692913	0.099977	-0.005562
N	0.770055	1.053885	-0.017172
S	3.386730	0.226183	0.014712
N	-2.042769	2.178586	-0.061186
H	1.593295	-2.012270	0.005830
H	-3.279439	-2.010863	0.035828
H	-2.947281	2.423331	0.291899
H	-1.286360	2.787433	0.190414

Radical (GAS)

Total Electronic Energy: -39.8364761306

H	-0.542000	0.929742	0.000459
H	-0.534181	-0.934256	0.000459
H	1.076181	0.004514	0.000459
C	-0.000000	0.000000	-0.000230

For the names of XYZ stationary geometries see Figure 10 in the main text. The protonation site is indicated in brackets.

Neutral form

Total Electronic Energy: -904.721364892

C	-1.939433	1.014090	0.002102
C	-0.720922	0.324627	0.002327
C	-0.800541	-1.057866	0.000659
N	-1.911582	-1.787832	-0.000683
C	-2.994068	-1.026444	-0.001488
N	-3.070932	0.300856	-0.000894
N	0.495156	-1.481602	-0.000673
C	1.276616	-0.359647	0.000012
N	0.588291	0.746138	0.001832
S	3.010235	-0.505410	-0.001445
C	3.466516	1.232672	0.000855
N	-2.015309	2.351287	0.023187
H	4.551516	1.250591	0.000715
H	3.092463	1.725191	0.891152
H	3.092200	1.727674	-0.887953
H	0.810369	-2.434280	-0.001804
H	-3.941772	-1.546902	-0.003843
H	-2.907816	2.792377	-0.079920
H	-1.189101	2.905393	-0.081404

Cation (N1)

Total Electronic Energy: -905.168318031

N	0.619821	0.745059	-0.000062
C	-0.677598	0.322192	-0.000200
C	-0.758849	-1.060387	-0.000048
N	0.524252	-1.482132	0.000145
C	1.312438	-0.361437	0.000045
N	-1.872543	-1.805588	0.000038
C	-2.953654	-1.098476	0.000030
N	-2.982913	0.259217	-0.000082
C	-1.874930	1.041362	-0.000039
N	-1.962327	2.354555	0.000042
S	3.038792	-0.505593	-0.000003
C	3.489664	1.234396	0.000009
H	4.574504	1.252128	-0.000413
H	3.115551	1.725695	0.890789
H	3.114854	1.725841	-0.890403
H	0.834310	-2.438163	-0.000066
H	-3.919147	-1.576722	0.000038
H	-2.837304	2.844190	0.000639
H	-1.118796	2.897049	0.000124
H	-3.891091	0.695790	-0.000002

Anionradical

Total Electronic Energy: -904.782123897

C	-2.033506	0.879889	0.051615
C	-0.745958	0.385926	-0.174939
C	-0.626797	-1.016354	-0.125058
N	-1.607832	-1.876644	0.115854
C	-2.788342	-1.269520	0.318758
N	-3.041405	0.025606	0.298504
N	0.671763	-1.266153	-0.383514
C	1.312989	-0.037871	-0.704844
N	0.428367	0.984032	-0.469507
S	3.024608	0.118328	-0.419634
C	3.236141	0.274383	1.393768
N	-2.297355	2.236514	0.083976
H	4.298021	0.411747	1.583807
H	2.896206	-0.623878	1.900959
H	2.691189	1.137812	1.763409
H	1.063047	-2.172774	-0.550629
H	-3.629706	-1.920599	0.514126
H	-3.256845	2.464312	-0.109516
H	-1.657570	2.787930	-0.461003

Neutral radical (N1)

Total Electronic Energy: -905.260418923

N	0.482734	0.923193	-0.365055
C	-0.747140	0.371392	-0.184484
C	-0.640894	-1.003588	-0.044170
N	0.681088	-1.273165	-0.143087
C	1.326623	-0.088759	-0.330104
N	-1.650564	-1.900630	0.162409
C	-2.808900	-1.315525	0.253292
N	-3.028096	0.000405	0.139151
C	-2.040730	0.959473	-0.162262
N	-2.397369	2.290159	0.068806
S	3.057133	0.005045	-0.525497
C	3.581838	0.447927	1.150198
H	4.661946	0.556866	1.112532
H	3.325512	-0.334555	1.856408
H	3.137952	1.391022	1.450101
H	1.116385	-2.175184	-0.077994
H	-3.686540	-1.916756	0.438366
H	-2.088402	2.902844	-0.667460
H	-2.049287	2.651906	0.948058
H	-3.971020	0.337877	0.217561

Transition State (N1)

Total Electronic Energy: -905.227236

C	0.000000	0.000000	0.000000
C	0.000000	0.000000	1.386544
C	1.273802	0.000000	2.019336
N	2.474433	-0.044219	1.421784
C	2.396011	-0.079905	0.113780
N	1.255297	-0.064377	-0.570255
N	1.009109	0.026980	3.318854
C	-0.370368	0.079253	3.502007
N	-0.981991	0.037954	2.298683
S	-1.079808	0.025876	5.025868
N	-1.061332	0.007620	-0.888757
C	-1.439428	-2.148702	5.529475
H	-1.879538	-2.038227	6.510776
H	-0.451451	-2.585113	5.512990
H	-2.113277	-2.487288	4.756655
H	1.685899	0.045995	4.060494
H	3.300874	-0.124544	-0.470562
H	-1.852290	0.511277	-0.522198
H	-1.376058	-0.918422	-1.151777
H	1.288423	-0.061201	-1.576094

Complex (N1)

Total Electronic Energy: -905.254806057

C	-1.480281	-0.904198	0.578087
C	-0.193191	-0.408180	0.389291
C	-0.035806	0.972672	0.389332
N	-0.997484	1.893351	0.548063
C	-2.174163	1.382376	0.715754
N	-2.431849	0.050984	0.733859
N	1.281721	1.159620	0.195667
C	1.892233	-0.081707	0.084407
N	0.991805	-1.047744	0.202259
S	3.570372	-0.231727	-0.167899
N	-1.792801	-2.188765	0.610555
C	-1.787588	0.047385	-3.265525
H	-0.754585	0.314163	-3.411314
H	-2.541883	0.815005	-3.228174
H	-2.066252	-0.987409	-3.159157
H	1.762676	2.038421	0.139618
H	-3.032266	2.018596	0.854306
H	-1.063365	-2.864871	0.487074
H	-2.728736	-2.518915	0.745037

H	-3.388501	-0.229558	0.878096

Neutral radical (C8)

Total Electronic Energy: -905.283076904

N	0.445700	0.977566	-0.812375
C	-0.644195	0.361319	-0.373344
C	-0.484834	-1.044344	-0.276387
N	0.762768	-1.301490	-0.683461
C	1.427597	-0.055650	-0.995504
N	-1.411196	-1.889253	0.134780
C	-2.557513	-1.280795	0.456276
N	-2.853338	0.007005	0.413131
C	-1.908675	0.865522	0.001136
N	-2.188116	2.168421	-0.043603
S	2.934461	0.224304	0.007212
C	2.228020	0.269614	1.660580
H	3.059391	0.394698	2.347809
H	1.720922	-0.662038	1.893963
H	1.547516	1.107886	1.771429
H	1.206771	-2.199774	-0.683588
H	-3.351125	-1.931293	0.796510
H	-3.094658	2.494205	0.228925
H	-1.500060	2.828263	-0.347902
H	1.806745	-0.050568	-2.018379

Complex (C8)

Total Electronic Energy: -905.165170

N	0.200853	-1.551334	0.013537
C	0.358351	-0.653774	0.979570
C	1.668675	-0.642808	1.518232
N	2.344795	-1.594120	0.862495
C	1.493409	-2.151934	-0.158717
N	2.089976	0.153359	2.481878
C	1.131563	0.969849	2.930365
N	-0.127217	1.065914	2.537214
C	-0.552103	0.260990	1.552389
N	-1.816719	0.347182	1.141652
S	2.121498	-1.927006	-1.861834
C	0.260794	4.304506	0.119617
H	1.064013	5.013181	0.230718
H	0.150746	3.499287	0.826446
H	-0.433281	4.401773	-0.698022
H	3.328836	-1.768099	0.936724
H	1.425669	1.642501	3.723875
H	-2.435942	1.007593	1.569262
H	-2.157450	-0.240817	0.407011
H	1.431012	-3.242212	-0.07886

b) Thermodynamic Thresholds

For the names of XYZ stationary geometries see Figures 5 and 6. The figure to which the structure refers is indicated in brackets.

ASCH3 (see Figure 6)

Total Electronic Energy: -904.828964

C	-0.009580	0.006449	0.031428
C	-0.006292	0.004895	1.431528
C	1.241643	-0.000965	2.034950
N	2.422211	-0.004948	1.423421
C	2.285198	0.000112	0.103274
N	1.158792	0.007111	-0.613599
N	0.974709	0.001080	3.383105
C	-0.394144	0.009372	3.513565
N	-1.021718	0.012226	2.371614
S	-1.119641	0.014884	5.102098
C	-2.852326	0.013815	4.595412
N	-1.156703	-0.012899	-0.682300
H	-3.432132	0.012987	5.514240
H	-3.064687	-0.875218	4.010231
H	-3.066374	0.903015	4.011133
H	1.654375	-0.003882	4.124831
H	3.203162	-0.000348	-0.471510
H	-1.100571	0.117641	-1.676373
H	-2.031836	0.122216	-0.209692

ASCH3$^{\bullet -}$ (see Figure 6)

Total Electronic Energy: -904.819057

C	0.003938	0.102180	-0.076291
C	-0.146033	0.355886	1.291387
C	0.965558	0.001210	2.085876
N	2.103450	-0.529530	1.652445
C	2.109242	-0.709895	0.315649
N	1.144043	-0.426777	-0.546748
N	0.606902	0.345500	3.347598
C	-0.659205	1.007924	3.289969
N	-1.139315	0.922267	2.013243
S	-1.644356	1.109668	4.703076
C	-2.494697	-0.546205	4.816554
N	-1.037903	0.369943	-0.967816
H	-3.186693	-0.504915	5.658203
H	-1.763673	-1.335838	4.976308
H	-3.039356	-0.718449	3.891570
H	1.219956	0.358503	4.142019
H	3.017382	-1.130158	-0.101633
H	-0.702299	0.590521	-1.893790
H	-1.672853	1.065708	-0.600262

TS_180a (see Figure 6)

Total Electronic Energy: -904.783693409

N	0.257538	1.217438	0.010538
C	-0.865117	0.427767	0.022428
C	-0.508944	-0.930453	-0.006649
N	0.858790	-1.007876	-0.051143
C	1.219985	0.307474	-0.028851
N	-1.399207	-1.936559	-0.022815
C	-2.651098	-1.518868	-0.006811
N	-3.119117	-0.258851	0.031053
C	-2.222834	0.726155	0.051889
N	-2.671235	2.031810	0.133067
S	2.906520	0.788393	-0.143326
C	3.699318	-0.774022	0.309167
H	4.772374	-0.592876	0.286697
H	3.430712	-1.545840	-0.407943
H	3.391350	-1.074502	1.307657
H	1.395965	-1.740061	-1.226688
H	-3.414776	-2.289738	-0.028103
H	-3.603387	2.165045	-0.223844
H	-1.991815	2.713637	-0.166505

complex_180a (see Figure 6)

Total Electronic Energy: -904.789582874

N	0.268867	1.203668	0.064410
C	-0.853022	0.417794	0.059398
C	-0.485224	-0.943824	0.048438
N	0.871170	-1.026755	0.050052
C	1.221672	0.271568	0.057165
N	-1.387088	-1.948677	0.025818
C	-2.636521	-1.529427	0.001850
N	-3.107674	-0.268491	-0.000580
C	-2.211120	0.715051	0.034750
N	-2.661189	2.025077	0.078018
S	2.916483	0.779132	0.022578
C	3.708627	-0.843529	-0.003490
H	4.782497	-0.668135	-0.031934
H	3.390440	-1.400763	-0.879837
H	3.439126	-1.407286	0.884976
H	0.313529	0.045507	-2.479706
H	-3.401381	-2.299931	-0.023861
H	-3.575324	2.149991	-0.326109
H	-1.967696	2.694933	-0.217463

m/z = 180a (see Figure 5)

Total Electronic Energy: -904.288288

N	-0.027059	0.025896	-0.001626
C	-0.013032	0.034164	1.370171
C	1.319298	-0.001594	1.835567
N	2.152679	-0.026839	0.765960
C	1.281677	-0.009444	-0.257362
N	1.635063	-0.018313	3.149042
C	0.581916	-0.008474	3.941523
N	-0.723977	0.018228	3.617188
C	-1.025281	0.046392	2.319790
N	-2.361783	0.118382	1.951522
S	1.921888	-0.036418	-1.906598
C	0.366641	0.015181	-2.821123
H	-0.188080	0.914279	-2.566892
H	-0.242023	-0.851383	-2.577326
H	0.783768	-0.027873	5.008547
H	-2.986490	-0.265631	2.642448
H	-2.530759	-0.193368	1.007577
H	0.621670	0.013055	-3.879206

m = 1 (see Figure 5)

Total Electronic Energy: -0.498206

H	0.000000	0.000000	0.000000

TS_166 (see Figure 6)

Total Electronic Energy: -904.814954295

N	0.388524	0.990366	-0.329984
C	-0.810026	0.384918	-0.097667
C	-0.700405	-1.013865	-0.056649
N	0.619911	-1.251440	-0.261742
C	1.274682	-0.014891	-0.459645
N	-1.699467	-1.873593	0.127286
C	-2.877733	-1.257937	0.269544
N	-3.129538	0.047963	0.249947
C	-2.098821	0.885682	0.073286
N	-2.333154	2.250154	0.095485
S	2.953409	0.065644	-0.640568
C	3.808849	0.284063	1.378071
H	4.869189	0.415774	1.181280
H	3.576820	-0.630809	1.912272
H	3.326726	1.166896	1.781412
H	1.073551	-2.144158	-0.330292
H	-3.736701	-1.903427	0.414897
H	-3.268368	2.506629	-0.177062
H	-1.598963	2.786833	-0.342012

complex_166 (see Figure 6)

Total Electronic Energy: -904.832937

N	0.913096	0.490728	-0.599415
C	0.153908	0.112311	0.478613
C	0.847973	-0.762556	1.318476
N	2.061348	-0.902262	0.720547
C	2.081004	-0.133275	-0.452272
N	0.376817	-1.317326	2.435775
C	-0.876905	-0.951004	2.686253
N	-1.657041	-0.134357	1.977635
C	-1.148056	0.413316	0.865957
N	-1.924564	1.293034	0.148741
S	3.463174	-0.103762	-1.446417
C	-0.796234	2.027398	-3.084916
H	-1.362690	1.423235	-3.775885
H	-0.993655	3.086860	-3.039582
H	0.010718	1.592398	-2.509930
H	2.839616	-1.461040	1.021112
H	-1.326580	-1.371320	3.579210
H	-2.915076	1.198489	0.297626
H	-1.629913	1.455420	-0.804102

m/z = 166 (see Figure 5)

Total Electronic Energy: -865.000617

N	-0.022969	0.030065	0.021025
C	-0.006845	0.035434	1.391413
C	1.295495	0.010717	1.899299
N	2.079801	-0.002351	0.789809
C	1.256683	0.005123	-0.348726
N	1.632718	-0.009441	3.189182
C	0.568173	-0.012591	3.986877
N	-0.721011	0.005526	3.645491
C	-1.021375	0.036880	2.341389
N	-2.345903	0.101887	1.964174
S	1.947976	-0.021029	-1.905094
H	3.082278	-0.027157	0.738205
H	0.771074	-0.036173	5.051972
H	-2.993498	-0.266603	2.640567
H	-2.511433	-0.159303	1.004363

m = 15 (see Figure 5)

Total Electronic Energy: -39.825355

C	0.384879	0.222210	0.093916
H	-0.083455	-0.048183	1.024652
H	1.085889	-0.449532	-0.370942
H	0.153638	1.165173	-0.370942

TS_16 (see Figure 6)

Total Electronic Energy: -904.710722152

N	0.524590	0.580913	-0.180955
C	-0.840592	0.242672	-0.143008
C	-0.961963	-1.131212	0.053807
N	0.327229	-1.625705	0.134957
C	1.152079	-0.534927	-0.011819
N	-2.096522	-1.809530	0.144591
C	-3.139123	-0.979806	0.021715
N	-3.145584	0.338605	-0.167249
C	-1.980922	1.060501	-0.266511
S	2.899450	-0.742207	0.044440
C	3.349352	0.998168	-0.124926
N	-0.901369	3.577576	0.425092
H	4.435418	1.040521	-0.099298
H	2.920053	1.568573	0.693207
H	2.969308	1.384462	-1.065599
H	0.599550	-2.579356	0.296391
H	-4.113813	-1.458654	0.086228
H	-1.486279	3.598091	-0.412443
H	-0.146832	2.956280	0.119875

complex_16 (see Figure 6)

Total Electronic Energy: -904.713577

N	-0.449699	0.268999	-0.587536
C	-0.559862	-0.237452	0.724146
C	0.714390	-0.558156	1.188742
N	1.592312	-0.248103	0.165141
C	0.812518	0.239535	-0.856850
N	1.007149	-1.059900	2.378623
C	-0.113730	-1.222177	3.094301
N	-1.372510	-0.954080	2.758862
C	-1.707467	-0.430019	1.523967
S	1.561251	0.754821	-2.364935
C	0.052257	1.254706	-3.220665
N	-3.692335	0.685009	-0.761175
H	0.356213	1.609649	-4.202252
H	-0.442504	2.043320	-2.661710
H	-0.618390	0.405208	-3.308507
H	2.590913	-0.359149	0.171691
H	0.039237	-1.632283	4.090669
H	-3.384645	0.292583	0.149854
H	-2.775712	0.766139	-1.216057

m/z = 16 (see Figure 5)

Total Electronic Energy: -55.871919

N	0.004905	0.000000	0.003959
H	-0.006856	0.000000	1.028378
H	1.003766	0.000000	-0.223743

m = 165 (see Figure 5)

Total Electronic Energy: -848.771341

N	-0.007298	0.002765	-0.012189
C	-0.028480	-0.001462	1.366639
C	1.280047	-0.003525	1.884648
N	2.091507	0.000646	0.781035
C	1.258973	0.004460	-0.315915
N	1.625441	-0.007865	3.162189
C	0.590911	-0.010236	3.986757
N	-0.701011	-0.009083	3.606869
C	-0.991190	-0.004932	2.358010
S	1.936930	0.010159	-1.921352
C	0.391061	0.012499	-2.856178
H	0.678364	0.015872	-3.903881
H	-0.183809	0.900476	-2.614439
H	-0.183147	-0.877392	-2.619975
H	3.098100	0.001720	0.778702
H	0.795035	-0.014116	5.048322

m/z = 180b (see Figure 5)

Total Electronic Energy: -904.254969

N	0.009794	-0.006748	-0.039591
C	-0.014887	-0.013323	1.351127
C	1.269632	-0.003960	1.854615
N	2.095351	0.008533	0.744997
C	1.268225	0.006040	-0.349227
N	1.664125	-0.006290	3.137987
C	0.581897	-0.019798	3.930568
N	-0.697525	-0.030190	3.626956
C	-1.114006	-0.027213	2.291599
N	-2.356949	-0.035789	1.936103
S	1.935886	0.019147	-1.980105
C	0.365992	0.012618	-2.870416
H	-0.212131	0.890609	-2.598420
H	-0.199732	-0.876029	-2.607116
H	0.804737	-0.023509	4.996722
H	-2.905740	-0.044484	2.794323
H	0.609002	0.019553	-3.930226
H	3.098994	0.017812	0.758652

m/z = 180c (see Figure 5)

Total Electronic Energy: -904.216527

N	-0.129435	0.037164	0.118908
C	-0.062086	0.034602	1.493383
C	1.255656	0.011061	1.947785
N	2.000453	0.006368	0.804866
C	1.124192	0.018319	-0.278598
N	1.647319	-0.015094	3.222224
C	0.615201	-0.025100	4.059225
N	-0.687601	-0.008784	3.770135
C	-1.042743	0.028483	2.480600
N	-2.373465	0.091081	2.156596
S	1.811830	-0.001588	-1.920227
C	0.614532	-0.004478	-3.160222
H	0.021501	0.907333	-3.164806
H	0.004997	-0.905373	-3.144059
H	3.002546	-0.017442	0.732588
H	0.859134	-0.054272	5.115304
H	-3.008970	-0.238746	2.862496
H	-2.591411	-0.137266	1.199680

m/z = 180d (see Figure 5)

Total Electronic Energy: -904.181724

N	-0.008277	0.030614	-0.052105
C	-0.014880	0.041974	1.344878
C	1.271233	0.000658	1.867179
N	2.085748	-0.030516	0.733056
C	1.253798	-0.011535	-0.354000
N	1.641053	-0.012963	3.129648
C	0.623022	0.005013	4.069635
N	-0.695069	0.039675	3.593731
C	-1.017416	0.065913	2.317368
N	-2.363625	0.148770	1.957716
S	1.923164	-0.045065	-1.982414
C	0.366696	0.037336	-2.890547
H	-0.165349	0.946135	-2.625431
H	-0.249644	-0.821681	-2.642747
H	-2.960834	-0.167634	2.707134
H	0.623281	0.034822	-3.947125
H	3.089658	-0.059708	0.735533
H	-2.576226	-0.256516	1.059350

Experimental

a) Synthesis of 8-bromoadenine

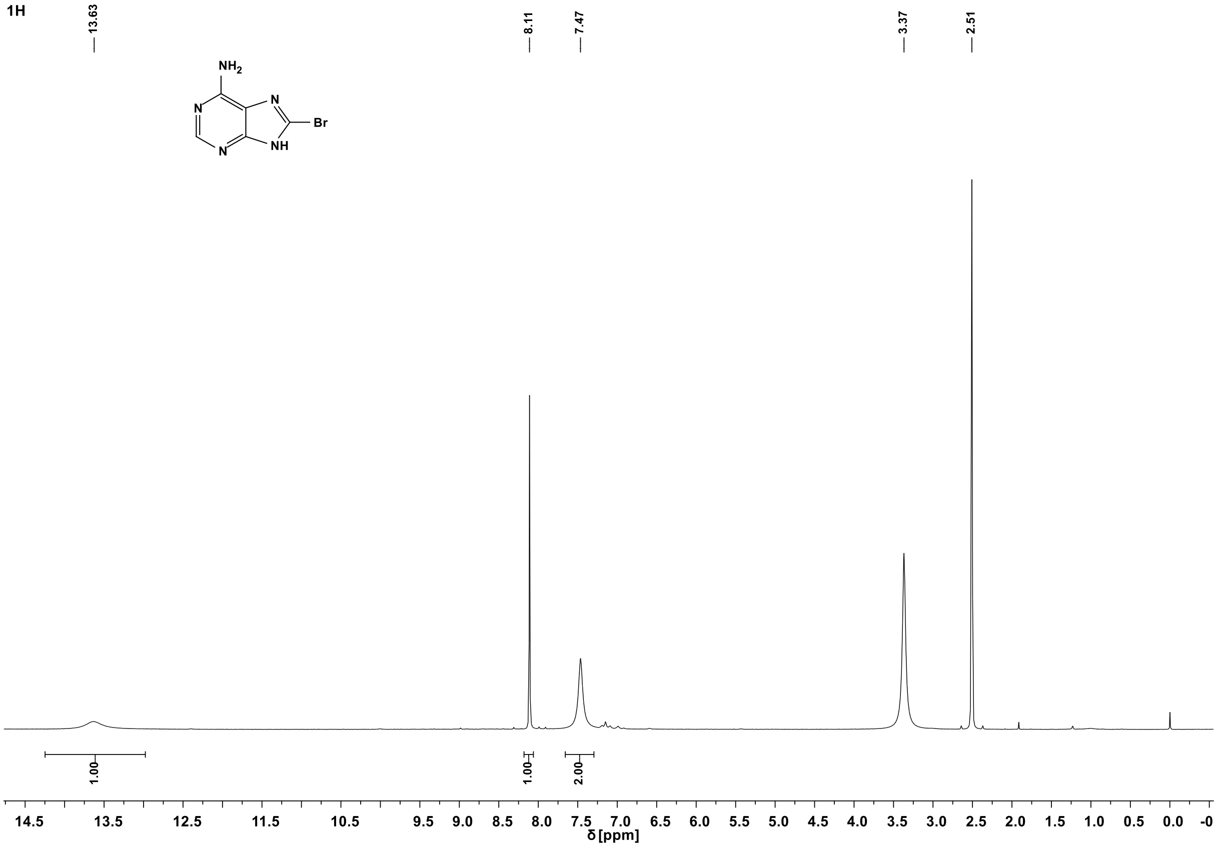

Figure S1. ^{1}H NMR spectrum of 8-bromoadenine.

b) Synthesis of 6-amino-7(*H*)-purine-8(9*H*)-thione

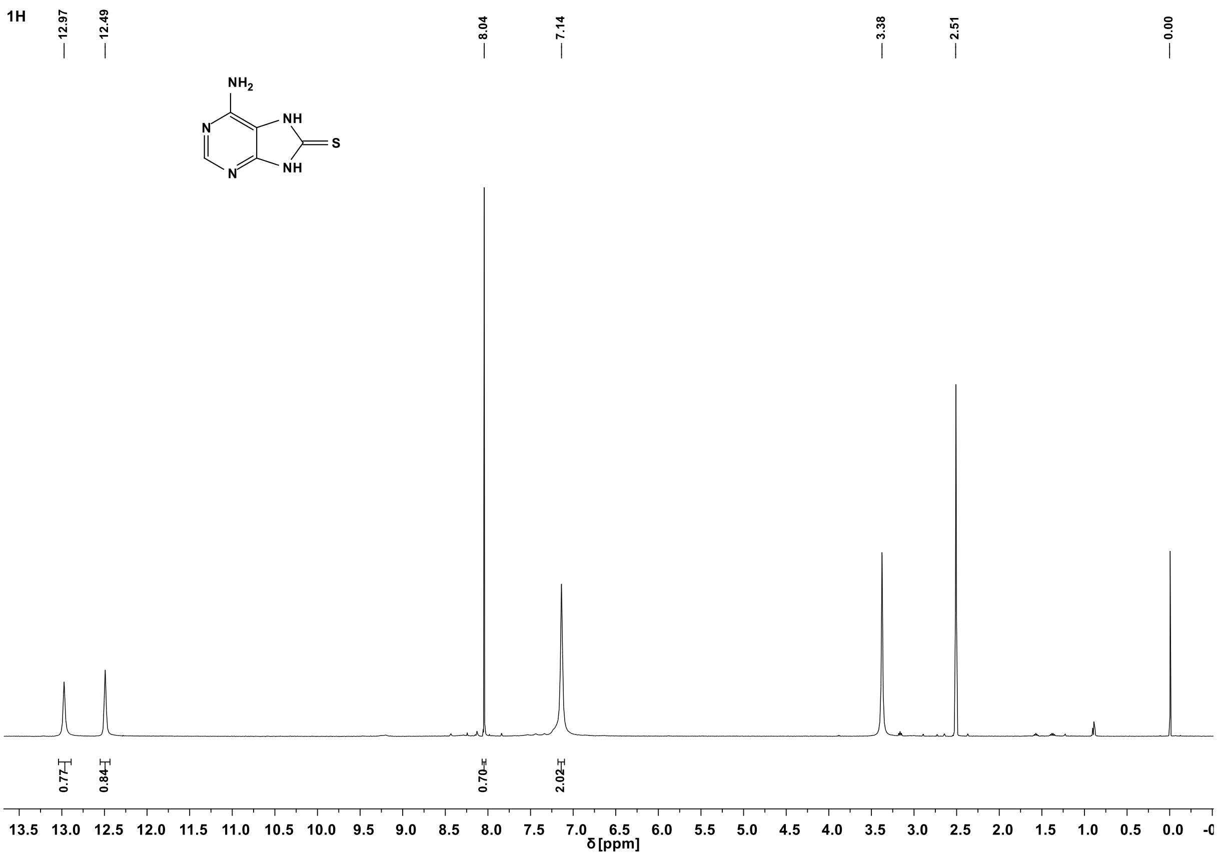

Figure S2. ^{1}H NMR spectrum of 6-amino-7(*H*)-purine-8(9*H*)-thione.

c) Synthesis of 8-thiomethyladenine

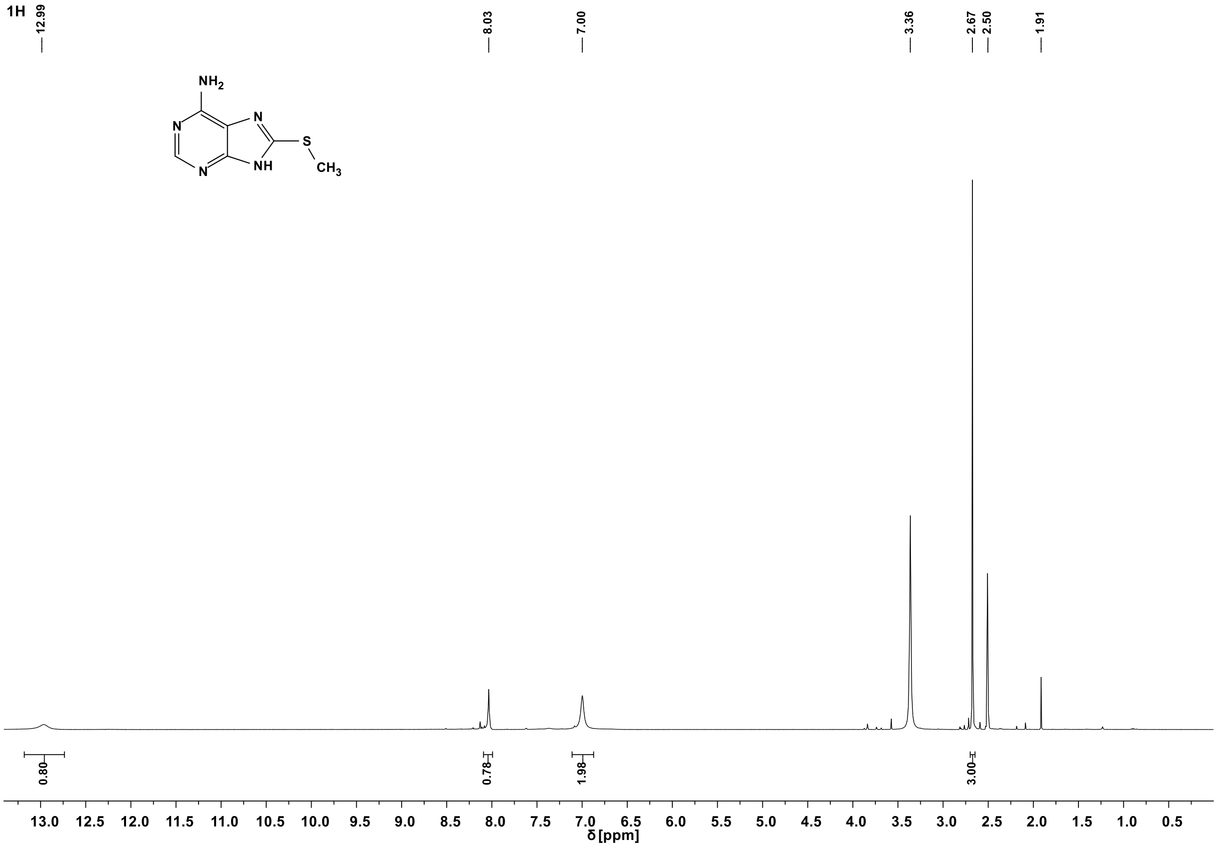

Figure S3. ^{1}H NMR spectrum of 8-thiomethyladenine.

d) HPLC analysis

HPLC Dionex UltiMate 3000 System with Diode Array Detector was used to analyse the irradiated and non-irradiated samples, which were separated by a Wakopak Handy ODS (4.6 x 150 mm, 5 μm in particle size and 100 Å in pore size) reverse-phase column and flow rate 1 mL·min^{-1}. The program of analysis was set to linear gradient 0-35% of phase B in 20 minutes (phase A: 0.1% formic acid in deionized water and phase B: 80% acetonitrile in deionized water).

e) LC-MS analysis

LC-MS analysis of synthesized product and radiolysis products have been recorded using a Nexera X2 Ultraperformance Liquid Chromatography (UHPLC) coupled to a Triple

TOF 5600+ tandem mass spectrometer (SCIEX), equipped with a duo-electrospray interface, operated in the negative ionization mode.

Chromatographic conditions: A C18 column (Kinetex Phenomenex; C18; 2.1×150 mm; 2.6 µm, 100 Å) was used for chromatographic separation with a flow rate equal 0.3 mL·min^{-1} and injection volume of 50 µL; the analysis program was set to linear gradient 0-35% of phase B for 20 minutes and 25°C; phase A: 0.1% HCOOH in deionized water, phase B: 80% ACN in deionized water. During analysis, the eluent was directed to waste for 1 min after injection.

MS and MS/MS conditions: the spray voltage was -4.5 kV, the source temperature was 300°C and the nebulizer gas (N_2) pressure was 25 psi.

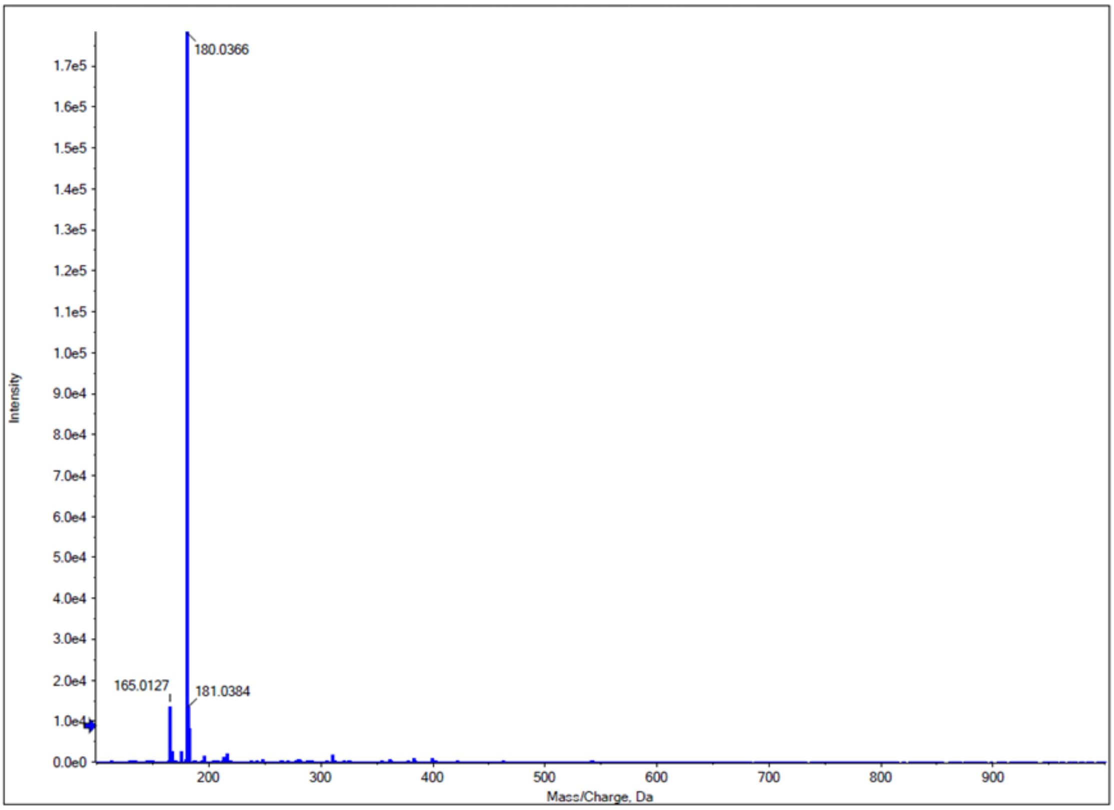

Figure S4. MS spectrum of 8-thiomethyladenine.

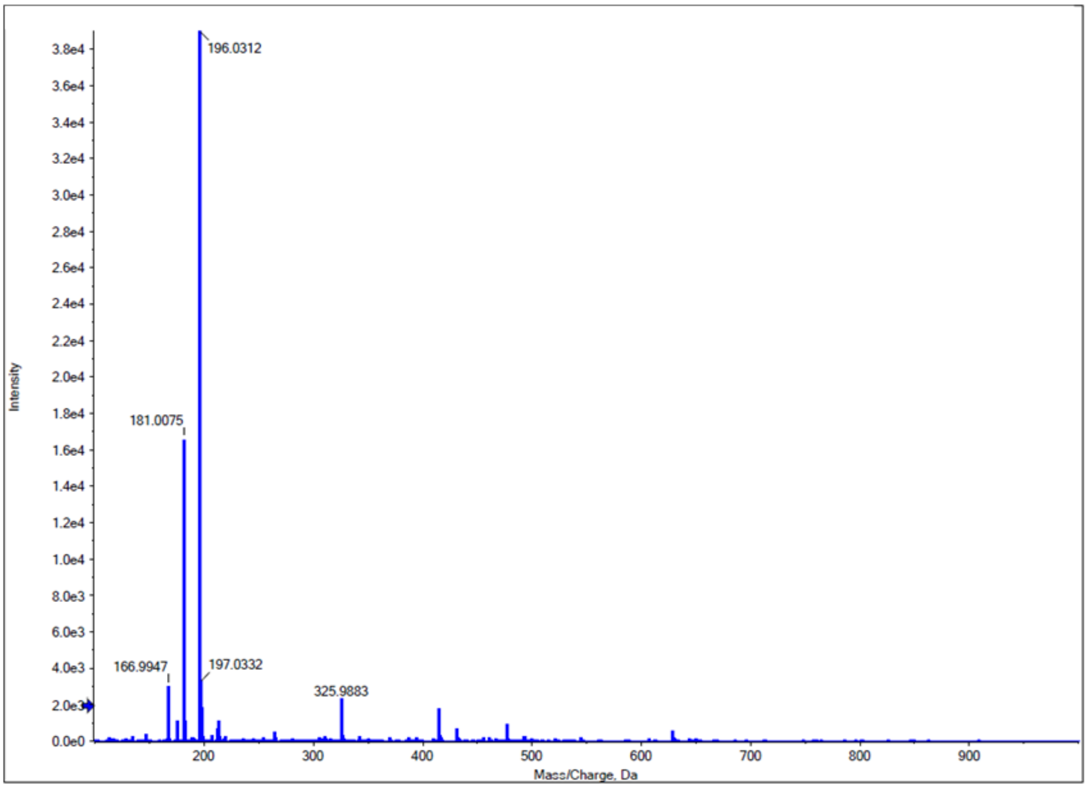

Figure S5. MS spectrum corresponding with a peak at 5.06 min (see Figure 9 in the main text).

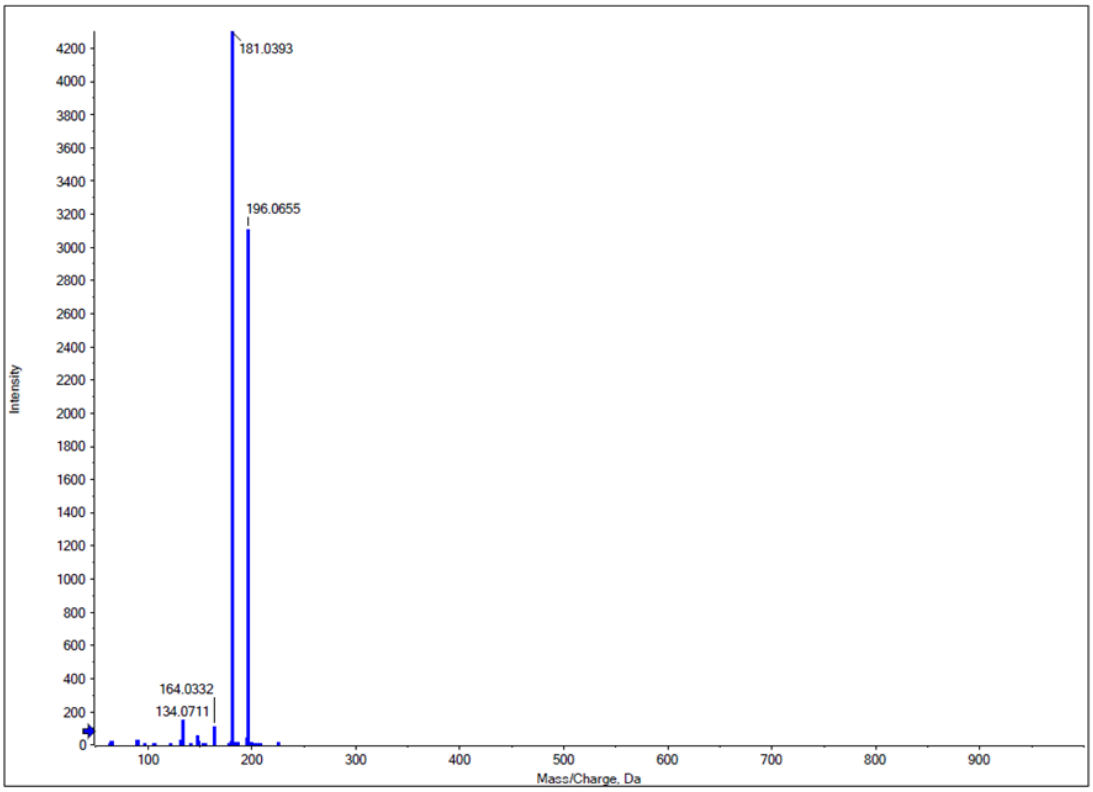

Figure S6. MS/MS spectrum corresponding with m/z equal 196 Da.

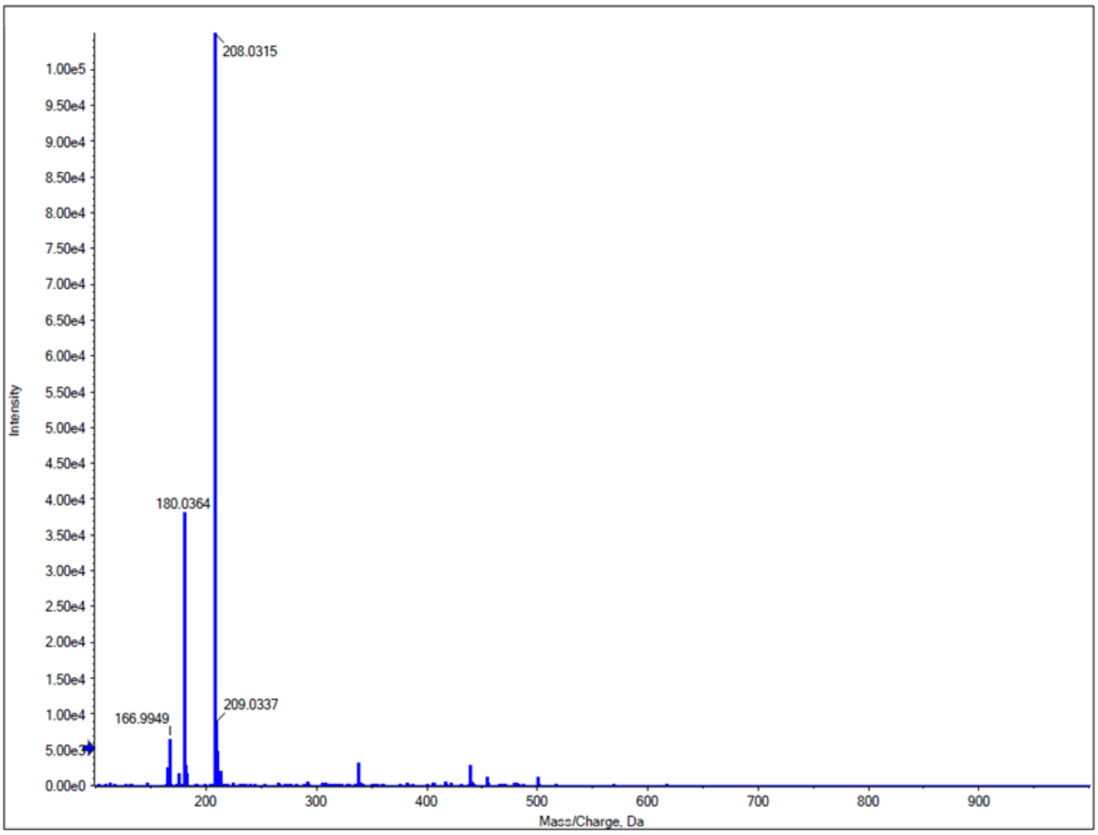

Figure S7. MS spectrum corresponding with a peak at 13.01 min (see Figure 9 in the main text).

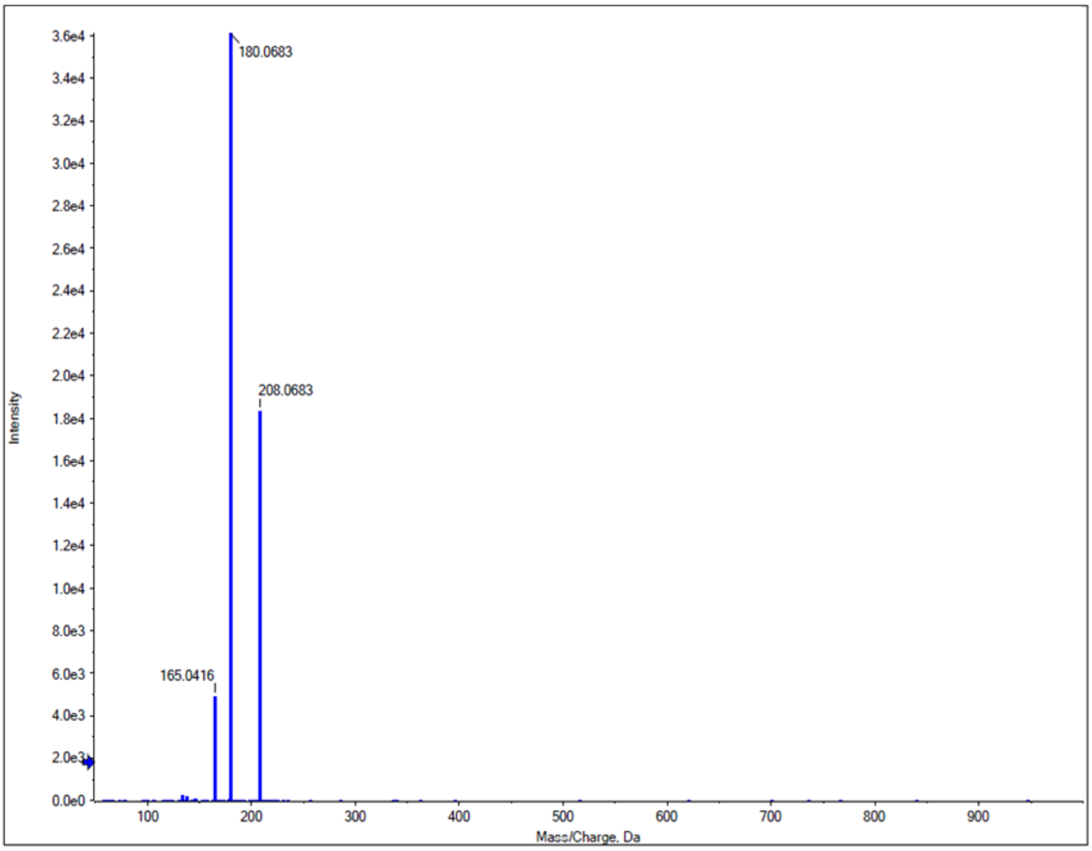

Figure S8. MS/MS spectrum corresponding with m/z equal 208 Da.
